# Stochastic block models reveal a robust nested pattern in healthy human gut microbiomes

**DOI:** 10.1093/pnasnexus/pgac055

**Published:** 2022-05-23

**Authors:** Sergio Cobo-López, Vinod K Gupta, Jaeyun Sung, Roger Guimerà, Marta Sales-Pardo

**Affiliations:** Departament d’Enginyeria Química, Universitat Rovira i Virgili, 40007 Tarragona, Catalonia, Spain; Microbiome Program, Center for Individualized Medicine, Mayo Clinic, Rochester, MN 55905, USA; Division of Surgery Research, Department of Surgery, Mayo Clinic, Rochester, MN 55905, USA; Microbiome Program, Center for Individualized Medicine, Mayo Clinic, Rochester, MN 55905, USA; Division of Surgery Research, Department of Surgery, Mayo Clinic, Rochester, MN 55905, USA; Departament d’Enginyeria Química, Universitat Rovira i Virgili, 40007 Tarragona, Catalonia, Spain; Institució Catalana de Recerca i Estudis Avançats, 08010 Barcelona, Catalonia, Spain; Departament d’Enginyeria Química, Universitat Rovira i Virgili, 40007 Tarragona, Catalonia, Spain

**Keywords:** stochastic block models, statistical inference, ecological networks, human microbiome, nestedness

## Abstract

A key question in human gut microbiome research is what are the robust structural patterns underlying its taxonomic composition. Herein, we use whole metagenomic datasets from healthy human guts to show that such robust patterns do exist, albeit not in the conventional enterotype sense. We first introduce the concept of mixed-membership enterotypes using a network inference approach based on stochastic block models. We find that gut microbiomes across a group of people (hosts) display a nested structure, which has been observed in a number of ecological systems. This finding led us to designate distinct ecological roles to both microbes and hosts: generalists and specialists. Specifically, generalist hosts have microbiomes with most microbial species, while specialist hosts only have generalist microbes. Moreover, specialist microbes are only present in generalist hosts. From the nested structure of microbial taxonomies, we show that these ecological roles of microbes are generally conserved across datasets. Our results show that the taxonomic composition of healthy human gut microbiomes is associated with robustly structured combinations of generalist and specialist species.

Significance Statement Is the gut microbiome a random ecosystem or does it have an internal structure? We show that gut microbial communities in healthy individuals have a well-defined and robust ecological order consisting of gradually increasing degrees of specialization: while some microbes are found in the majority of humans, others are very specific to some people. Similarly, hosts have varying degrees of microbial diversity. Our findings describe the principles of how healthy human gut microbiomes are associated with robustly structured combinations of generalist and specialist species.

## Introduction

Among the different communities of microbes that live in symbiosis inside and on the human body, the gut microbiome has received the most attention by far (1–3). During the last decade, gut microbiome dysbiosis was found to be linked to a variety of diseases related to the gut (1, 4–6), and even to those not overtly gut-related, including fertility disruption (7), neurological pathologies (8), or atopy and asthma in infants (9–11). Recent studies continue to show that the gut microbiome plays an important role in health and disease, such as influencing immune response in infants (11) and regulating inflammation (12).

The need to better understand why the gut microbiome is such an important component in human health has sent scientists on a quest to discover “healthy” gut microbiome compositions. Such an understanding is critical for the design of better therapies based on fecal microbiota transplantation (FMT) (13, 14) or gut microbiome-targeting drug treatments (3). Despite recent progress, the scientific community has yet to reach a consensus that pinpoints the essential microbial components for our well-being. By and large, this has been due to the large variability observed in gut microbiome composition and that only few species are present in the majority of samples (15–18). This can be partially explained by an ecological perspective, in which the occupation of niches determines the governing functions of the ecological system (15). From birth, the microbial niches of the gastrointestinal tract are affected by many dietary and environmental factors, resulting in much of the observed variability (2, 19, 20).

Despite their known heterogeneity in taxonomic composition from person to person, gut microbiomes from healthy subjects are able to perform nearly the same critical biological functions (21–23); this suggests that there must be recurrent patterns in the structure of the human gut microbiome that are linked to the healthy phenotype. An approach to characterize and classify such patterns is the identification of so-called enterotypes, defined by specific traits of microbiome composition profiles that are common to particular groups of hosts. However, despite some consistencies in the most abundant species, which suggests that there are taxa more preferred than others, enterotypes have not been found to be convincingly robust across, for example, geographically distinct populations (23). As a result, the search for robust, rigid classification criteria of microbiome compositions (such as the original concept of enterotypes) has been somewhat abandoned in favor of more nuanced studies aiming to understand the taxonomic and functional landscape of the gut microbiome and its connection to various pathologies (23).

The fundamental assumption behind the definition of enterotypes is that there are groups of hosts that have similar microbe abundance profiles. On the other hand, there is a class of generative models called bipartite stochastic block models (SBMs) (24), which (for microbiome research purposes) explicitly assume the existence of groups of hosts and groups of microbes that are associated according to similarities in the microbe abundance profiles. These statistical models can be seen, in practice, as a biclustering technique that allows simultaneous clustering of both microbes and hosts. Additionally, they are easily interpretable and are amenable to rigorous and unbiased model selection tools to identify optimal groupings of hosts and microbes (25). In fact, SBMs have been successfully used in the complex networks literature to characterize the large-scale structure of complex networks and to make predictions of unobserved data (26–28).

In our study, we represent gut microbe abundance profiles from a cohort of healthy people as a network and follow a Bayesian SBM approach to show: first, that the SBMs are able to predict unobserved microbe abundances better than agglomerative hierarchical clustering methods; and second, that there exists a robust organization structure in healthy gut microbiomes based on taxonomic composition. Our analysis reveals that microbe abundances display the same nested structure that was first observed in faunas of fragmented habitats and archipelagos (29), and later discovered in a wide range of ecological systems (30–33).

In this nested structure of gut microbiomes, we find that a few groups of hosts have significant abundances of a large number of microbial groups (generalist hosts), while other groups of hosts have very few microbial groups of significant abundance (specialist hosts). Analogously, some microbial groups are present in nearly all groups of hosts (generalist microbial groups), while other microbial groups are present in just a few groups of hosts (specialist microbial groups).

Our results suggest that the taxonomic composition of healthy human gut microbiomes follow a nested structure, which are not only similar to those found in other ecological networks throughout nature, but also predictive of unobserved abundances. By showing that there are generalist hosts as well as specialist hosts, and that there is a varying gradient of such ecological roles across different hosts (in contrast to discrete enterotypes), we mathematically formulate a new description of how gut microbiomes organize within a small population.

## Materials and Methods

### Downloading and quality control of sequenced reads

The current investigation uses publicly available human gut microbiome datasets from five published studies (Table 1). Of note, for studies whose cohort includes microbiome samples from both cases (disease) and controls (healthy), only samples from the controls were considered. Raw sequence files (.fastq) were downloaded from the NCBI Sequence Read Archive (SRA) and European Nucleotide Archive (ENA) databases. Sequence reads were processed with the KneadData quality-control pipeline, which uses Trimmomatic v0.36 and Bowtie2 v0.1 for removal of low-quality read bases and human reads, respectively. Trimmomatic v0.36 was run with parameters SLIDINGWINDOW:4:30, and the Phred quality score threshold was set at “<30”. Illumina adapter sequences were removed, and trimmed nonhuman reads shorter than 60 bp in nucleotide length were discarded. Potential human contamination was filtered by removing reads that aligned to the human genome (reference genome hg19). Furthermore, stool metagenome samples of low read count after quality filtration (< 1 M reads) were excluded from our analysis.

**Table 1.** Description of healthy human gut microbiome datasets used in this study.

Author	Study title	No. of microbial species	No. of healthy subjects
W. Liu *et al*.	Unique features of ethnic mongolian gut microbiome revealed by metagenomic analysis (37)	128	107
N. Qin *et al*.	Alterations of the human gut microbiome in liver cirrhosis (38)	137	92
M. Schirmer *et al*.	Linking the human gut microbiome to inflammatory cytokine production capacity (39)	134	467
C. Huttenhower *et al*., J. Lloyd-Price *et al*.	Structure, function and diversity of the healthy human microbiome (16), Strains, functions and dynamics in the expanded human microbiome project (40)	118	222
D. Zeevi *et al*.	Personalized nutrition by prediction of glycemic responses (41)	144	883

### Species-level taxonomic profiling

Taxonomic profiling was done using the MetaPhlAn2 v2.7.0 phylogenetic clade identification pipeline (34) using default parameters. Briefly, MetaPhlAn2 classifies metagenomic reads to taxonomies based on a database of clade-specific marker genes derived from ≈17,000 microbial genomes (corresponding to ≈13,500 bacterial and archaeal, ≈3,500 viral, and ≈110 eukaryotic species). After taxonomic profiling, the following stool metagenome samples were discarded from our analysis: (i) samples composed of more than 5% unclassified taxonomies; and (ii) phenotypic outliers according to a dissimilarity measure. More specifically, Bray–Curtis distances were calculated between each sample of a particular phenotype and a hypothetical sample in which the species’ abundances were taken from the medians across samples. A sample was considered as an outlier, and thereby removed from further analysis, when its dissimilarity exceeded the upper and inner fence (i.e., > 1.5 times outside of the interquartile range above the upper quartile and below the lower quartile) of all dissimilarities. Finally, species of viral origin, those of either unclassified or unknown clades, and those of low prevalence (i.e., observed in < 1% of the samples in each study), were excluded from our study.

### Discretization of microbiome datasets

Each gut microbiome dataset of a study can be represented as a species matrix **M**, wherein columns represent gut microbiome samples of the hosts and rows correspond to microbial species. Each element *M_hm_* corresponds to the relative abundance (i.e., proportion) of microbial species *m* in the sample of host *h*. Importantly, because our interest was to find the leading patterns in microbiome composition, we only considered as nonzero the relative abundances that were larger than 10^−4^. In addition to matrices of species-level abundances, we considered abundance matrices of phylum, class, order, family, and genus taxonomic ranks.

We model species abundance matrices as bipartite multilink networks, i.e. networks with two classes of nodes (hosts and microbes) in which edges run between nodes of different classes and edges are of different types. Here, edge weights correspond to relative abundances of microbes in each host. Since relative abundances are continuous and our models require a discrete number of edge types, we need to discretize the relative abundances and then define a new species matrix $\hat{\bf M}$ in which entries $\widehat{M}_{hm}$ are categorical. We choose to discretize the microbe abundances according to their order of magnitude. In all five datasets, abundances ranged from 10^−4^ to 10^−1^, and so we considered three types of edges (categorical abundances): null or negligible, $\widehat{M}_{hm}=\rm I$ if *M_mh_* < 10^−4^; low, $\widehat{M}_{hm}=\rm II$ if 10^−4^ ≤ *M_mh_* ≤ 10^−3^); and high, $\widehat{M}_{hm}= \rm III$ if *M_mh_* > 10^−3^. Despite the known limitations associated with discretizing data, representing the abundance data by a small set of classes provides a simpler and more straightforward interpretation of “microbial abundance” without significantly altering the inherent distribution of the compositional data.

### SBMs for microbe abundance matrix modeling and enterotype identification

We define a bipartite network with categorical edges whose weights are defined by the microbial relative abundances. This bipartite network is the starting point toward our overall aim, which is to find underlying groups of both microbes and hosts (essentially two classes of nodes) that together best explain (data-grounded) host–microbiome relationships.

Our bipartite SBMs assume that hosts can be classified into groups that connect similarly to the same microbes, that is, that have the same relative abundances of the same microbes. At the same time, we assume that microbes can also be classified into groups that have similar relative abundances in similar groups of hosts. Furthermore, to each host and microbe group pairs (*h, m*), we assign a set of probabilities $p_{hm}=\lbrace p_{hm}^{\rm I}, p_{hm}^{\rm II}, p_{hm}^{\rm III}\rbrace$, which correspond to the probability that a host in group *h* has negligible, low, or high relative abundances of microbes in group *m*. Note that in conventional enterotype identification approaches, each group of hosts would define a so-called enterotype.

**Fig. 1.**
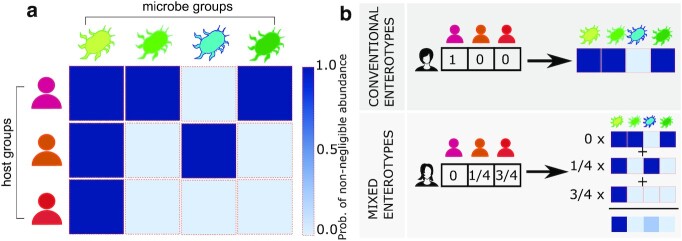
SBMs provide a theoretical framework to model microbe abundance patterns. SBMs assume the existence of groups of hosts and groups of microbes. Here, we illustrate the main concept of SBMs in the context of microbe abundance matrix modeling and mixed-membership enterotype identification. (a) Probabilistic matrix of abundances. Each row corresponds to a group of hosts and each column to a group of microbes. Hosts in the same group display a similar microbe abundance pattern (row); microbes in the same group display similar relative abundance patterns across groups of hosts (column). For simplicity, we illustrate the case in which only two possible relative abundances are allowed: non-negligible (relative abundance ≥ 10^−4^) and negligible (relative abundance < 10^−4^). Colors represent the probability that a group of microbes is present in a group of hosts with non-negligible relative abundances (dark blue, *p*(non-negligible) → 1; light blue, *p*(non-negligible) → 0). (b) Relationship between SBMs and enterotypes. In single-membership SBMs, each host belongs to a host group exclusively (hot pink in the example), so that the corresponding (hot pink) row of the probability abundance matrix (in (a)) indicates the most likely abundances of each microbe group in hosts of that host group. Therefore, single-membership SBMs are equivalent to conventional enterotypes. In mixed-membership SBMs, each host belongs to a mixture of host groups with a certain probability given by her membership vector. The enterotype of each host is then a weighted combination of underlying enterotypes in the probabilistic matrix of abundances in (a) as depicted in the weighted sum of matrix rows. The latter model is more expressive and is better suited to model complex data (28, 35).

If there are *L* groups of microbes, then each host group *h* is assigned a vector of sets of probabilities for all the *L* microbe groups, **p**_*h*_ = (*p*_*h*1_, *p*_*h*2_,..., *p_hM_*). We call **p**_*h*_ enterotype, since the vector of probability sets determines the most likely set of microbe abundances for those hosts in group *h* in the same way that conventional enterotypes do (Fig. 1).

#### Mixed-membership stochastic block models for enterotype modeling and identification

To increase the expressibility of our model, we consider a bipartite mixed-membership stochastic block models (MMSBMs) (28, 35) in which we allow for real hosts and microbes to belong to all of the groups with a finite probability (Fig. 1). In this case, the groups are no longer associated to a group of hosts or microbes and, therefore, they become *latent* groups, that is underlying groups that we cannot directly measure. Each latent group of hosts is then characterized by its corresponding latent enterotype. Therefore, a possible interpretation is that the observed relative microbe abundances for hosts are the result of mixing underlying enterotypes.

Formally, we model the multiedge bipartite network defined by categorical species matrices $\widehat{{\bf M}}$ as a bipartite MMSBM. MMSBMs assume there exist *K* latent groups of hosts and *L* latent groups of microbes. Each host *h* belongs to host group *k* with probability θ_*hk*_ and each microbe *m* belongs to group *l* with probability η_*ml*_. These probabilities are encoded in $\boldsymbol{\theta }_h$ and $\boldsymbol{\eta }_m$, the so-called mixed-membership vectors for host *h* and microbe *m*:

(1) $$\begin{eqnarray*}
\boldsymbol{\theta }_h=\left(\theta _{h1}, \theta _{h2}, \dots , \theta _{hK} \right) \qquad \boldsymbol{\eta }_m=\left(\eta _{m1}, \eta _{m2}, \dots , \eta _{mL} \right)~~.
\end{eqnarray*}
$$

Because θ_*h*_ and η_*m*_ are probability vectors, they are subject to the normalization condition that all probabilities have to add to one,

(2) $$\begin{eqnarray*}
\sum ^{K}_{k=1}\theta _{hk}=1 \qquad \sum ^{L}_{l=1}\eta _{ml}=1 ~.
\end{eqnarray*}
$$

Note that if single membership SBMs are the best description for observed abundance matrices, then we will find that hosts have a large probability of belonging to only one group.

If ${\cal A}:=\lbrace \rm I,\rm II,\rm III\rbrace$ is the set of possible values each edge can take, MMSBMs further assume that the probability that microbes in group *m* have relative abundance $a\in {\cal A}$ in hosts of group *h* is *p*(*a*)_*k*ℓ_. Since for each pair of groups (*k*, ℓ) each abundance can only take one value, these probabilities are also subject to the normalization condition

(3) $$\begin{eqnarray*}
\sum _{a\in {\cal A}} p(a)_{k\ell } =1~~~\forall (k,\ell ).
\end{eqnarray*}
$$

Note that **p** is a tensor of dimensions *K* × *L* × *A*, with $A\equiv |{\cal A}|$. Each ’row’ *k* in this tensor defines the latent enterotype for group *k*. If there are only two abundance types *A* = 2, and because of the normalization condition we only need one matrix **p** of dimensions *K* × *L* to define the model as we illustrate in Fig. 1.

Given the mixed-membership vectors $\boldsymbol{\theta }$ and $\boldsymbol{\eta }$ and the probability tensor **p**, the MMSBM defines the probability that microbe *m* has abundance of type *a* in host *h* as

(4) $$\begin{eqnarray*}
{\rm Pr}[\widehat{M}_{mh}=a] = \sum _{k, \ell } \theta _{hk}\, p_{k \ell } (a) \, \eta _{m\ell } .
\end{eqnarray*}
$$

#### Inference for model parameter estimation

Given a categorical species matrix $\widehat{\bf M}^O$ of observed microbial abundances of *M* microbial species in *P* hosts, the MMSBMs assumes that each relative abundance observation is independent from the others. Therefore, the probability of observing $\widehat{\bf M}^O$ according to the model is given by the following likelihood function,

(5) $$\begin{eqnarray*}
p(\widehat{\bf M}^O |{\rm MMSBM})=p(\widehat{\bf M}^O |\boldsymbol{\theta },\boldsymbol{\eta }, \mathbf {p}) =\prod _{(h,m)\in \widehat{\bf M}^O } {\rm Pr}[\widehat{M}^O_{hm}].
\end{eqnarray*}
$$

Note that the product runs over all observed abundances. If some abundances are missing (i.e. not observed), then these terms do not appear in the likelihood.

If we assume that we have flat priors over model parameters, then we can obtain the set of parameters $(\boldsymbol{\theta }^{*},\boldsymbol{\eta }^{*}, \mathbf {p}^{*})$ that best describe the observed data by maximizing the likelihood

(6) $$\begin{eqnarray*}
\{\boldsymbol{\theta }^{*},\boldsymbol{\eta }^{*}, \mathbf{p}^{*}\} = \displaystyle\mathop{{\rm arg\,\, max}}_{\{{\boldsymbol\theta },\,{\boldsymbol\eta },\,{\bf {p}}\}}\,\, \{p\,(\widehat{\bf M}^{o}|{\boldsymbol\theta },\,{\boldsymbol\eta },\,{\bf{p}})\} .
\end{eqnarray*}
$$

#### Equations for the Expectation Maximization algorithm

We follow a variational approach to obtain iterative equations for the model parameters and use an Expectation Maximization algorithm to obtain estimates for $\lbrace \boldsymbol{\theta }^{*},\boldsymbol{\eta }^{*}, \mathbf {p}^{*}\rbrace$ (28). Specifically, we find for the model parameters $\boldsymbol{\theta }, \boldsymbol{\eta }, \mathbf {p}$ iterative equations that depend on auxiliary probability distributions as it is usual in Expectation Maximization approaches.

In particular, we find the following equations (28):

(7) $$\begin{eqnarray*}
\theta _{hk}& = & \frac{1}{d_h} \bigg [ \sum _{a \in \lbrace \rm I,\rm II,\rm III\rbrace } \sum _{m \in \partial _h^{a}} \sum _{\ell } \omega _{hm}(k,\ell ) \bigg ] \, ,
\end{eqnarray*}
$$

(8) $$\begin{eqnarray*}
\eta _{m\ell }& =& \frac{1}{d_{m}} \bigg [\sum _{a \in \lbrace \rm I,\rm II,\rm III\rbrace } \sum _{h \in \partial _m^{a}} \sum _{k} \omega _{hm}(k,\ell ) \bigg ] \, ,
\end{eqnarray*}
$$

(9) $$\begin{eqnarray*}
p_{k\ell }(a)& =& \frac{\sum _{ [(h,m) \in R^o|a_{hm}=a]} \omega _{hm}(k,\ell )}{\sum _{(h^{\prime },m^{\prime }) \in R^o} \omega _{h^{\prime }m^{\prime }}(k,\ell )} \, ,
\end{eqnarray*}
$$

where $\partial _m^{a}=\lbrace h |\widehat{M}_{mh}=a\rbrace , \, a \in \lbrace \rm I,\rm II,\rm III\rbrace$, and $\partial _h^{a}=\lbrace m|\widehat{M}_{mh}=a\rbrace , \, a \in \lbrace \rm I,\rm II,\rm III\rbrace$. *d_h_* and *d_m_* are the degrees of host *h* and microbe *m* in the bipartite representation of $\widehat{M}$, that is, the number of microbes with measured relative abundance reported for host *h*, and the total number of hosts for which the relative abundance of microbe *m* has been reported, respectively.

In the expressions above, ω_*pm*_(*k*, ℓ) is an auxiliary distribution that appears in the variational approach used to maximize likelihood functions using Expectation Maximization algorithms (28). This distribution represents the probability that host *h* having the microbe *m* with relative abundance *a_hm_* is due to *h* and *m* belonging to latent groups *k* and ℓ, that is

(10) $$\begin{eqnarray*}
\omega _{hm}(k,l)=\frac{\theta _{hk} \eta _{m\ell } p_{k,\ell }(a)}{\sum _{k^{\prime }\ell ^{\prime }}\theta _{hk^{\prime }} \eta _{m\ell ^{\prime }} p_{k^{\prime }\ell ^{\prime }}(a)} .
\end{eqnarray*}
$$

#### Selection of *K* and *L*

We assume that the best model is the most predictive one. We, therefore, look for the combination of model parameters *K* and *L* that maximizes the predictive accuracy (see section on metrics and Figure S1 in Supplementary Material). In order to be consistent, we test our results on the combination of all five datasets. We find that the most predictive combination of parameters corresponds to *K* = 10, *L* = 20, that is 10 latent enterotypes and 20 latent groups of microbes (see Figure S2, Supplementary Material)

### Host-specific cross-validation experiments

For each microbe abundance profile of host *h*, we randomized the order of abundances and partitioned the profile into five equally sized groups (folds) for cross-validation. In each of the cross-validation experiments for host *h*, we used as the training data for the model (cluster identification or model parameter estimation) all other microbe abundance profiles (columns of the species matrix **M**) that do not correspond to host *h* along with four of the five-folds from host *h*. After model training, we used the (parameter) fitted model to make predictions on the abundances of the fold held out during training (see Supplementary Methods for further details). We performed this process for all five folds in each and every host.

#### Predictions in cross-validation experiments

##### SBM approach

For each missing microbial abundance, we have three different scores associated to the probability that host *h* has microbe *m* with null, low or high abundance: ${\rm Pr}[a_{hm}=\rm I]$, ${\rm Pr}[a_{hm}=\rm II]$, and ${\rm Pr}[a_{hm}=\rm III]$. Our estimation in each case is given by the maximum of these probabilities, i.e.

(11) $$\begin{eqnarray*}
\rm Prediction = \rm \max \limits _x \lbrace \rm Pr[a_{hm} = x] \rbrace \,.
\end{eqnarray*}
$$

We, thus look for the membership vectors $\boldsymbol{\theta }$ and $\boldsymbol{\eta }$ that yield the highest predictive power of unobserved abundances.

##### Conventional approach

The agglomerative clustering pipeline we consider rigorously selects the best out of 90 possible clusterings. For each host *h* and each fold, we select the best clustering to estimate unobserved abundances. We first consider all the hosts that belong to the same cluster as host *h*. Then, for each missing microbe *m_i_*, we estimate its relative abundance as the most likely abundance of that microbe in the other hosts within the cluster.

### Similarity distance between membership vectors of microbes according to taxonomic classification

Suppose we have a taxonomic level *T* with *t*_1_, …, *t_q_* taxa. For each taxa *t_k_*, we take all possible pairs of microbes’ membership vectors $(\eta ^i_k, \eta ^j_k)$, such that *i, j* ∈ *t_k_*, and *i* ≠ *j* and compute the Euclidean distance *d_ij_*(*t_k_*) between them. Note that the smaller the distance, the more similar the membership vectors of the two microbes. We repeat this process for all *n* taxa and then compute the mean intrataxa distance as

(12) $$\begin{eqnarray*}
\langle d_{{\it intra}} \rangle =\frac{1}{N^P_{{\it intra}}}\sum ^q_{k=1} \sum _{i \ne j} d_{ij}(t_k) \ ,
\end{eqnarray*}
$$

where $N^P_{{\it intra}}$ is the total number of pairs of microbes from the same taxa. Next, we measure the mean intertaxa distance. To that end, we take all possible pairs of membership vectors from different taxa $(\eta ^i_k, \eta ^j_{k^{\prime }})$, such that *k* ≠ *k*^′^ and measure the Euclidean distances between them. We repeat this process for all pairs of different taxa and calculate the mean:

(13) $$\begin{eqnarray*}
\langle d_{{\it inter}} \rangle =\frac{1}{N^P_{{\it inter}}}\sum ^q_{k \ne k^{\prime }} \sum _{i,j} d_{ij}(t_k, t_{k^{\prime }}) \ .
\end{eqnarray*}
$$

Finally, we take the log of the ratio intra/inter distances log (〈*d_intra_*〉/〈*d_inter_*〉) for all five taxonomic levels.

### Assessment of nestedness

In order to measure the nestedness of a binary matrix in which elements are either 1s or 0s, we use the metric presented in (36):

(14) $$\begin{eqnarray*}
n=\frac{ \sum _{i\lt j}q^{(h)}_{ij} + \sum _{i\lt j}q^{(m)}_{ij}}{\sum _{i\lt j} {\rm min}( q^{(h)}_{i}, q^{(h)}_{j}) + \sum _{i\lt j} {\rm min}( q^{(m)}_{i}, q^{(m)}_{j} )} \ ,
\end{eqnarray*}
$$

where *q_ij_* represents the number of shared interactions between rows/columns *i* and *j* and *q_i_* is the number of interactions of row/colum *i. h* and *m* represent rows (hosts) and microbes(columns), respectively. The numerical value of the nestedness *n* ranges from 0 to 1.

### Genome sizes and protein-coding gene counts for microbial species

Genome sizes and protein coding genes for all strains available in NCBI were retrieved for the species found to be present in the five datasets mentioned above. For each species, median genome size and median count of protein-coding genes were calculated from all of its strains (see Tables S1-3, Supplementary Material).

## Results

### SBMs are predictive of microbe abundances and can identify biologically relevant groups

The most common approaches for enterotype identification rely on clustering heuristics to identify groups (clusters) of hosts who share commonalities in gut microbiomes (23, 42, 43) (the exception being approaches based on multinomial mixture models (44, 45)). In contrast, we use SBMs as generative models to identify group patterns in microbe abundance profiles. SBMs first assume that there are distinct groups of hosts and groups of microbes, and that the probability that a host has a significant abundance of a certain microbe depends exclusively on the groups to which the host and the microbe belong (24). These generative models have only a few underlying assumptions (i.e., that there are groups of microbes and groups of hosts, and that links connecting a microbe group to a host group are independent of other links), and can be used to find the most plausible group memberships of hosts and of microbes that best describe/compress a given dataset. The most plausible group memberships derived from observable data are, by definition, also the most predictive of unobserved data.

SBMs are fully defined by the group memberships of hosts and microbes; and probability matrices $\mathbf {p}(a)$, whose elements *p*_*k*ℓ_(*a*) determine the probability that microbes of group ℓ are present with an abundance of *a* in hosts of group *k*. In this model, each row (for *k*) in the matrices $\mathbf {p}(a)$ describes the most likely abundances of each group of microbes in host group *k*, and therefore, is akin to what has been typically referred to as enterotypes (Fig. 1).

The same concept can be further generalized to allow hosts and microbes to simultaneously belong to multiple groups at varying probabilities (which increases the expressiveness of the model (28, 35)); and this is the type of SBM we consider in our analysis (see Fig. 1 and Methods for a formal definition of the model). SBMs have the additional advantage that they are amenable to efficient and well-grounded inference methods that can be used to optimally divide hosts and microbes into groups (27, 28) (see Methods).

To show how well SBMs can identify group patterns in microbe abundance matrices compared to previously demonstrated clustering approaches (e.g. for enterotype detection), we assess each model’s ability to predict relative abundances of unobserved microbes using five published gut microbiome datasets (Table 1). To this end, we first discretize the relative abundances *r* (which span a simplex distribution) into three discrete categorical bins: negligible (*r* < 10^−4^), low (10^−4^ ≤ *r* < 10^−3^), and high (*r* ≥ 10^−3^). The merit of this discretization technique is that it simplifies our prediction task into a classification problem. Then, we perform a host-specific five-fold cross-validation for all hosts within each dataset. Here, our main goal is to predict abundance categories of unobserved microbes in individual hosts as a way to test whether it would be possible to reconstruct the entirety of partially observed microbial abundance profiles. More specifically, we devised a cross-validation strategy that splits a profile of a given host into five equally sized partitions. For each split, we train a model with 80% (four of five partitions) of the microbe abundance profile of that host and with the entire abundance profiles of the remaining hosts; the held out 20% of the profile (from the same host) was used as the test set to evaluate predictive performance. The average prediction accuracy found in this manner provides us a means to objectively and quantitatively compare the robustness of group patterns identified by the SBM and other clustering approaches (25, 26, 46).

**Fig. 2.**
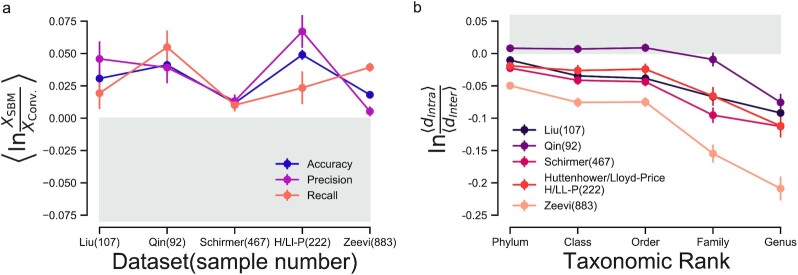
SBMs accurately predict and describe microbe abundances. (a) Predictive performance in host-specific cross-validation experiments. We compare the predictive power of SBMs and conventional approaches for enterotype identification using a cross-validation strategy centered on individual hosts (Methods). For each of the five different datasets indicated by the last name of the first author (number of samples), we compute the average log-ratio (over multiple folds) of the performance in predicting unobserved microbial abundances by SBMs to that by the conventional clustering approach (X: accuracy, precision, and recall—see Supplementary Material for definitions). Note that, in our prediction experiments of unobserved microbe abundances, the performance across different hosts is highly variable (see Figure S3, Supplementary Material): accuracy values are in the range of [0.5, 1], while recall and precision values have a wider range of [0.25, 1]. We compute average log-ratios, which allows us to measure performance differences between methods for the same prediction experiment. Note that a log-ratio equal to zero means that both methods have the same performance; a log-ratio above zero means that the SBM performs better than conventional heuristic approaches, and *vice versa*. Bars represent the standard error of the mean. (b) Similarity in microbe abundances across taxonomic ranks. For each of the five datasets, we compute the average distance between group membership vectors of microbes in the same taxa ('intra' taxonomic ranks), and compare it to the average distance between membership vectors of microbes in different taxa ('inter' taxonomic ranks; Methods and Table S1, Supplementary Material). Log-ratios smaller than zero indicate that the average intrataxa distance is smaller than the average intertaxa distance, that is, that membership vectors of microbes within a taxon are more similar than membership vectors of microbes across different taxa. Bars represent the standard error of the mean.

To compare against the predictive performance of the SBM approach, we use an agglomerative hierarchical clustering method described in (42), as such methods are generally regarded to be more reliable than divisive clustering (e.g., k-means) in providing accurate prediction models. Of note, the clustering method generates 90 different clusterings and then selects the best one based on multiple metrics. In our prediction experiments, we clearly see that the predictive performance of the SBM approach is systematically higher than that of the conventional clustering approach (Fig. 2a). Hence, we can conclude that SBMs are a superior predictive model.

Our analysis also shows that the majority of hosts and microbes are predictable; that is, that we are able to correctly predict unobserved abundances of microbes across many hosts, and also unobserved abundances in specific hosts for many microbes. Nonetheless, we also observe some variability in host and microbe predictability—some hosts and microbes have very strong patterns and are, therefore, more predictable than others (Figures S4–S8, Supplementary Material). Further analysis of the SBM groups reveals that the similarity of group memberships increases as we go down the taxonomic rank hierarchy (Fig. 2b). In particular, we find that the genus taxonomy has the largest intrataxon to intertaxon similarity ratio (Fig. 2b), suggesting that SBMs can infer biologically relevant groups in gut microbiomes.

### SBMs reveal the nested organization of microbe abundances in the human gut microbiome

Our results show that SBMs enable us to reasonably predict unobserved microbe abundances, indicating that our approach correctly models relationships between hosts and microbes in the five gut microbiome datasets. The questions we explore next are: What are these patterns? Are they consistent across datasets? Do these patterns reveal universal traits in the organization of gut microbiome ecologies? To these ends, we analyze the $\mathbf {p}$ matrices, which describe the abundances of groups of microbes in groups of hosts, for each of the five datasets.

**Fig. 3.**
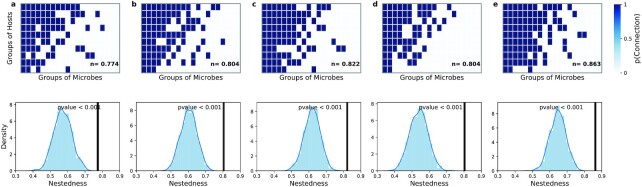
SBMs reveal underlying nested organization of microbe abundance matrices. ((a)–(e)) Nestedness of SBM $\mathbf {p}$ matrices for all datasets: (a) W. Liu *et al*., (b) N. Qin *et al*., (c) M. Schirmer *et al*., (d) C. Huttenhower *et al*. and J. Lloyd-Price *et al*., and (e) D. Zeevi *et al*. Top row: heatmaps represent SBM $\mathbf {p}$ matrices. Rows correspond to host groups, and columns correspond to microbe groups. As in ecological studies (see for instance (47)), we consider two categories of microbe relative abundance *r*: negligible ($\rm 0$, for *r* < 10^−4^) and non-negligible ($\rm 1$, for *r* ≥ 10^−4^), and analyze the corresponding SBM $\mathbf {p}$ matrices. Furthermore, because in this case the values $p_{k\ell }(a=\rm 1)$ matrices are very close to either 0 or 1, we round their values. Note that because our optimal choice of parameters is *K* = 10 groups of hosts and *L* = 20 groups of microbes, $\mathbf {p}$ matrices are 10 × 20 (see Methods). Dark blue matrix elements represent the presence of a microbe group in a host group with non-negligible abundance. Rows and columns are ordered according to their count of non-negligible abundances to show the nested organization (see Figure S9, Supplementary Material). The inset in each plot shows the numerical value of the nestedness (see Methods). Bottom row: significance of nestedness for the corresponding heatmap shown in the top row. For each dataset, we show the distribution of nestedness values obtained for 1,000 randomizations of the $\mathbf {p}$ matrix. For this, we randomize each $\mathbf {p}$ matrix while preserving the average number of connections between groups of hosts and microbes. The black solid line represents the nestedness value of the observed (actual) $\mathbf {p}$ matrix.

If we rank-order host groups according to their number of microbe groups of non-negligible relative abundances *r* (*r* ≥ 10^−4^), and rank-order microbe groups in an analogous manner, we find that biclustering $\mathbf {p}$ matrices display, what is called in ecology, a nested organization (29, 30, 32, 48) (Fig. 3; see Methods and Figure S9 (Supplementary Material) for an illustration). Such a nested organization entails that we can observe generalist and specialist groups of hosts and microbes. Generalist host groups have non-negligible abundances of the majority of microbes groups, while specialist host groups have non-negligible abundances of very few microbe groups. Importantly, microbe groups present in specialist hosts are subsets of those observed in generalist hosts; and thereby the nested structure. Moreover, microbe groups display the same behavior: generalist microbe groups have non-negligible abundances in the majority of host groups, while specialist microbe groups have non-negligible abundances in a few host groups. The nested structure entails that specialist hosts have non-negligible abundances of mainly generalist microbe groups, while specialist microbe groups are only present in generalist hosts (see Figure S9, Supplementary Material). Note that in our analysis, generalists and specialists are defined purely based on what we observe in the data, i.e. their positions within the nested hierarchy of the SBM $\mathbf {p}$ matrices. Therefore, these terms are not defined using the definition of generalist in other contexts.

**Fig. 4.**
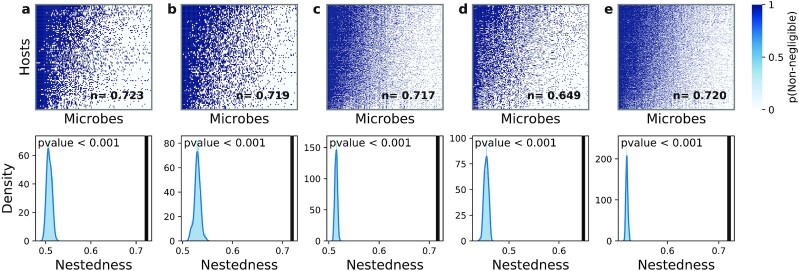
Microbe abundance matrices display a nested organization of hosts and microbes ((a)–(e)) Nestedness of microbe abundance matrices from all of the datasets considered: (a) W. Liu *et al*., (b) N. Qin *et al*., (c) M. Schirmer *et al*., (d) C. Huttenhower *et al*. and J. Lloyd-Price *et al*., and (e) D. Zeevi *et al*. Top row: categorical matrices with two categories of microbial species relative abundance *r*: negligible ($\rm 0$, for *r* < 10^−4^, white) and non-negligible ($\rm 1$, for *r* ≥ 10^−4^, blue). Rows represent individual hosts, and columns represent individual microbial species. Microbial species and hosts have been ordered according to the number of non-negligible abundances in their corresponding columns and rows, revealing a nested pattern (see Figure S9, Supplementary Material) as previously seen for the host and microbe groups. The inset in each plot shows the numerical value of the nestedness (see Methods). The bottom plots show the nestedness of 1,000 randomizations of the datasets. Black solid lines represent the observed (actual) nestedness value in the host-microbe abundance matrices above. Randomizations preserve the average number of connections between hosts and microbes.

Strikingly, we find that the nested organization in the SBM $\mathbf {p}$ matrices is also present in the original host–microbe abundance matrices (Fig. 4). That is, if we order hosts and microbial species by their degree (the number of non-negligible abundances in their corresponding rows and columns), the resulting ordered relative abundance matrices also display a nested structure (Fig. 4), allowing us to again identify generalists and specialists. Importantly, our results do not depend on the threshold we use to define non-negligible and negligible relative abundances (see Figure S10, Supplementary Material); even after changing the relative abundance threshold to below what was originally considered as negligible/non-negligible, nested hierarchies in the microbe abundance matrices are still observed.

### Ecological roles of microbial species are robust

**Fig. 5.**
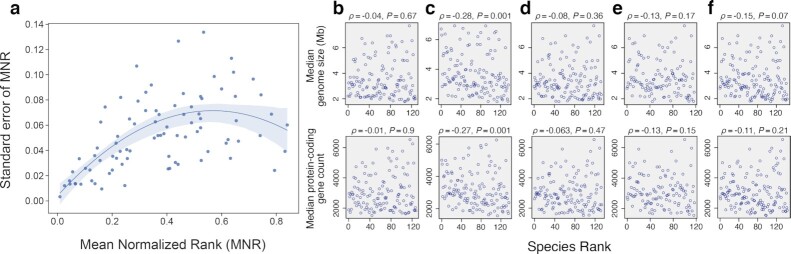
Ecological roles of microbial species are robust but not related to genome size or number of protein-coding genes. (a) Generalist and specialist roles are conserved across datasets. To every microbial species in a nested relative abundance matrix (Fig. 4), we assign a rank equal to the position of the column occupied by that microbial species. Low rankings correspond to generalist species, while high rankings correspond to specialist species (see Table S2 (Supplementary Material) for normalized ranks of each species in each dataset). For each microbe present in at least four of our datasets, we compute the mean normalized rank in the ordered abundance matrices in Fig. 4 and the standard error of the mean normalized rank. (We normalize the rankings to control for the different number of microbial species in each dataset.) The standard error tends to be smaller for species with low (generalists) and high (specialists) normalized rank, thus showing that generalist and specialized microbial species are conserved across datasets. In fact, the variance in normalized ranks across datasets is small for the vast majority of species, which implies that microbes tend to occupy the same position within the nested hierarchy. The line is a quadratic fit to the data and serves to guide the eye. (b)–(f) Relationship between genome size (top) and protein-coding gene count (bottom) with respect to the rank of a microbial species (see Methods and Tables S2 and S3, Supplementary Material). At the top of each panel, we show Spearman’s ρ and its corresponding *P*-value. There is no general trend in the datasets (except for the smallest dataset, panel (c)). This indicates that generalist and specialist species in nested matrices do not have obvious differences in genome size or the number of protein-coding genes. Study datasets: (b) Liu *et al*.; (c) Qin *et al*.; (d) Schirmer *et al*.; (e) Huttenhower *et al*. and J. Lloyd-Price *et al*.; and (f) Zeevi *et al*.. Points in the scatter-plots indicate individual microbiome samples.

In ecology, generalist species are considered to have a wide dietary breadth (i.e. species that can survive from a variety of food resources) and are less affected by environmental perturbations (49), whereas specialists have a narrower dietary breadth and can exhibit a competitive fitness advantage over generalist species within the confines of their habitat (50, 51). We now investigate whether microbial species in the gut can play the same roles. Indeed, we find that extreme roles (i.e. extreme generalists and specialists) are well conserved—generalist and specialist species play similar ecological roles in all datasets (Fig. 5a). Specifically, we find that generalist species concentrate in the *Clostridiales* and *Bacteroidales* orders (*Firmicutes* and *Bacteroidetes* phylum, respectively; see Tables S1 and S2, Supplementary Material), which is coherent with what enterotype identification studies have found to drive enterotype formation (23). Many specialist species, such as *Anaerotruncus colihominis* or *Cenarchaeum symbiosum*, are also well-conserved across datasets. A large part of these conserved specialist species belong to the *Firmicutes* phylum, including many in the *Clostridiales* order (Tables S1 and S2, Supplementary Material).

To investigate whether the ecological role of microbial species in the nested hierarchy (i.e. generalists and specialists) could be connected to their potential functional capacity, we looked at the relationship between genome sizes (and also number of protein-coding genes) of microbes and the ranks of microbes in the rank-ordered abundance matrices in Fig. 4. Here, low and high rank correspond to generalist and specialist microbes, respectively. We find no robust relationship between genome size (Fig. 5 b–f top row) and ecological role, or between the number of protein-coding genes and ecological role (Fig. 5 b–f bottom row; see Figure S11, Supplementary Material). This suggests that the ecological role microbes play in a nested organization may be independent of the range of functions a given species could potentially perform, but rather related to the occupation of the specific habitat.

### Generalist and specialist hosts show varying gut microbiome diversity

**Fig. 6.**
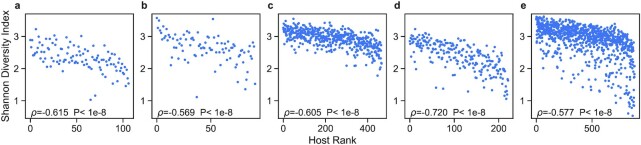
Relationship between Shannon diversity index and host rank. For each dataset, we show the Shannon diversity index vs. the rank of each host in the ordered relative abundance matrix (i.e. the position of the row which corresponds to a host in the ordered abundance matrix). Low ranks correspond to generalist hosts and high ranks correspond to specialist hosts. We also show the Spearman’s ρ and its corresponding *P*-value. In all datasets, we observe a significant inverse correlation between the Shannon diversity index and host rank. Study datasets: (a) Liu *et. al*, (b) Qin *et al*.l, (c) Schirmer *et al*., (d) Huttenhower *et al*. and Lloyd-Price *et al*., and (e) Zeevi *et al*. Points in the scatter-plots indicate individual gut microbiome samples.

Island biogeography studies show that a consequence of the nested structure in fragmented habitats and archipelagos is wide disparities in species richness across habitats (29, 48). For individual hosts, microbiome composition is often quantified in terms of a single alpha-diversity index (52). These diversity indicators have often been regarded as a possible indicator of gut health—the higher the diversity index, the healthier the host (3). Our results show that, within the nested organization of gut microbiome datasets, there is a significant correlation between the host’s rank (occupied row number) in the rank-ordered nested abundance matrix in Fig. 4 and that host’s Shannon diversity index. More specifically, generalist hosts have a higher diversity than specialist hosts (Fig. 6), confirming our earlier finding that generalists hosts provide a habitat for a wider range of gut microbes. In all, we conclude that ecological nestedness, which has been reported in past works in macroecology, extends to the human gut microbiota. Further examinations into this property could lead to new discoveries regarding the ecological assembly of the human gut microbiome.

## Discussion

Ever since links between gut microbiome dysbiosis and complex, chronic diseases were established, there has been a keen interest in understanding what a healthy gut microbiome would look like, and how therapies can be designed to restore gut dysbiosis to a healthy state. Fuelled by these interests, scientists have aimed to uncover recurrent patterns in gut microbiome composition by categorizing gut microbiomes into enterotypes. However, despite some broadly shared properties (18, 23), no universally recognized taxonomic property in the gut microbiomes of healthy subjects has been found. In this study, we demonstrate that gut microbiomes can indeed show patterns of organization that are common across different studies. Notably, these patterns are defined in terms of the ecological organization when considering both hosts and microbes simultaneously rather than hosts or microbes separately.

We used SBMs to formally identify robust organization principles of the healthy human gut microbiome. By focusing on both groups of microbes and hosts simultaneously, we found that microbe abundance matrices display a nested structure that elucidates well-defined ecological roles (i.e. generalists vs. specialists). Some hosts and microbial species are generalists: generalist microbes are present in non-negligible abundances in most hosts, while generalist hosts contain the majority of microbial species in non-negligible abundances. In contrast, some other hosts and microbes are specialists: specialist microbes are only present in generalist hosts, and specialist hosts only have non-negligible abundances of generalist microbes.

There has been much interest in the origin of nested ecologies, as such nested organization has been observed in a number of ecological systems (30). It was first reported in faunas of insular fragmented habitats and in archipelagos (29, 48). Other cases of nested hierarchies were found in endoparasite communities in fish (31) and parasite communities in geographically dispersed species of bats (33). Furthermore, nestedness has been found in ecosystems with mutualistic interactions (30, 32, 53, 54), i.e. ecological relationships benefiting both parties. Pollinator–plant networks are a common example of mutualistic networks; pollinators obtain their food while pollinizing the plants. Such nested ecological structures have even been suggested to play a role in optimizing and balancing species abundances in the presence of mutualistic interactions (55). Despite much progress in its study, however, the relationship between mutualism, nestedness, stability, and species diversity is still relatively poorly understood and remains a subject of close and careful investigation (36, 56–58).

We can establish several connections between human gut microbiomes and other nested ecosystems. First, the relationship between hosts and their microbiomes is well-known to be mutually beneficial. A prominent example is in food digestion, in which microbes in the gut, such as those in the *Bacteroidetes* phylum, aid in the degradation of dietary complex polysaccharides (15, 59–61). Second, we can draw straightforward analogies with findings in the macroecology literature. For the case of island biogeography, hosts can be viewed as islands and microbiota as the island fauna. Several mechanisms have been put forward to explain the nestedness observed in island faunas, such as selective extinction, colonization ability, habitat nestedness, and human intervention (30, 47, 62, 63). In regard to the gut microbiome, all of these explanations can offer promising clues and insights into how the microbes assemble and respond to perturbations. For instance, Cook and Quinn (47) found that, in collections of islands, fauna with large dispersal ability (such as birds) show more nested abundance patterns across islands than reptiles and mammals. Their finding could possibly suggest that, in a gut microbiome context, a gradient in colonization ability (in which dispersal ability is a strong factor) may contribute to the nested organization in microbial species abundances observed in our study.

Other studies have shown that when colonizing new patches, animals with larger trophic breadths (food generalists) build the first successful colonies; and thereafter, food specialists (typically predators that feed on a narrow range of prey) arrive at a later stage (64, 65). An explanation of these observations is that species with large trophic breadths are able to survive comparatively well in ecosystems subject to fluctuations, whereas food specialists need more mature, developed, and diverse ecosystems to survive. By drawing a direct analogy with these observations, our results suggest that specialist hosts have gut environments that offer relatively restricted living conditions for microbes. In summary, our study offers the opportunity to bridge the gap between macroecology and gut microbiome studies, and thereby shines a new perspective on human-associated microbial ecology research.

As a methodological point, we note that inferring clusters (or microbial communities) based on Dirichlet multinomial processes or other probabilistic mixture models is indeed a common approach for microbial community detection from metagenomic data (whether the inferred distributions are biologically meaningful or accurate is a different issue) (44, 45). To clarify, the Dirichlet multinomial mixture model pipeline developed by Holmes *et al*. (45) was designed to find clusters of microbial communities, whereas our SBM framework is a biclustering technique that allows simultaneous clustering of both microbes and hosts; in addition, the Dirichlet model was not designed for the predictive tasks we used to validate our approach, namely, prediction of held-out microbial relative abundances. Specifically, as part of its input, the Dirichlet model needs all the metagenomic reads in each host’s microbiome sample to give the inferred multinomial model parameters. However, its current implementation does not work when a subset of the microbial species’ reads are held-out in cross-validation, as we had done to compare our SBM approach against the agglomerative clustering pipeline. While it would be possible to program a different implementation of the Dirichlet model specifically intended for our prediction experiments, this task falls outside of the scope of our current study.

To conclude, we demonstrate that the human gut microbiome does indeed have robust structural patterns underlying its taxonomic composition. To this point, our study may contribute toward defining the design principles of a healthy gut microbiome, developing better models to understand gut microbiome evolution, and establishing strategies to restore or maintain wellness in the form of probiotic communities. An open question is whether diseases can disrupt the nested structure of healthy microbiomes. If that were the case, nestedness would be a straightforward metric of the health state of gut microbiomes, and interventions could be designed to restore the original ecological pattern. Explorations into these topics will be the subject of our future studies.

## Supplementary Material

pgac055_Supplemental_Files

## Data Availability

All data needed to evaluate the conclusions of this study are present in the manuscript and Supplementary Materials. Code for the inference using mixed-membership stochastic block models can be found at https://github.com/SergioCoboLopez/Repository_Enterotypes.

## References

[1] Byrd AL , Segre JA. 2016. Adapting Koch’s postulates. Science. 351(6270):224–226. 26816362 10.1126/science.aad6753

[2] Singh RK , et al. 2017. Influence of diet on the gut microbiome and implications for human health. J Trans Med. 15(1):73. 10.1186/s12967-017-1175-y PMC5385025 28388917

[3] Gilbert JA , Lynch SV. 2019. Community ecology as a framework for human microbiome research. Nat Med. 25(6):884–889. 31133693 10.1038/s41591-019-0464-9 PMC7410146

[4] Mohajeri MH , et al. 2018. The role of the microbiome for human health: from basic science to clinical applications. Eur J Nut. 57(1):1–14. 10.1007/s00394-018-1703-4 PMC5962619 29748817

[5] Zuo T , Ng SC. 2018. The gut microbiota in the pathogenesis and therapeutics of inflammatory bowel disease. Front Microbiol. 9:2247. 30319571 10.3389/fmicb.2018.02247 PMC6167487

[6] Durack J , Lynch SV. 2019. The gut microbiome: relationships with disease and opportunities for therapy. J Exp Med. 216(1):20–40. 30322864 10.1084/jem.20180448 PMC6314516

[7] Qi X et al. 2019. Gut microbiota–bile acid–interleukin-22 axis orchestrates polycystic ovary syndrome. Nat Med. 25(8):1225–1233. 31332392 10.1038/s41591-019-0509-0 PMC7376369

[8] Sharon G , Sampson TR, Geschwind DH, Mazmanian SK. 2016. The central nervous system and the gut microbiome. Cell. 167(4):915–932. 27814521 10.1016/j.cell.2016.10.027 PMC5127403

[9] Arrieta MC , et al. 2015. Early infancy microbial and metabolic alterations affect risk of childhood asthma. Sci Trans Med. 7(307):307ra152–307ra152. 10.1126/scitranslmed.aab2271 26424567

[10] Avershina E , et al. 2016. Transition from infant- to adult-like gut microbiota. Environ Microbiol. 18(7):2226–2236. 26913851 10.1111/1462-2920.13248

[11] Durack J , et al. 2018. Delayed gut microbiota development in high-risk for asthma infants is temporarily modifiable by Lactobacillus supplementation. Nat Commun. 9(1):707. 29453431 10.1038/s41467-018-03157-4 PMC5816017

[12] Blander JM , Longman RS, Iliev ID, Sonnenberg GF, Artis D. 2017. Regulation of inflammation by microbiota interactions with the host. Nat immunol. 18(8):851–860. 28722709 10.1038/ni.3780 PMC5800875

[13] Xiao Y , Angulo MT, Lao S, Weiss ST, Liu YY. 2020. An ecological framework to understand the efficacy of fecal microbiota transplantation. Nat Commun. 11:3329. 32620839 10.1038/s41467-020-17180-x PMC7334230

[14] Smillie CS , et al. 2018. Strain tracking reveals the determinants of bacterial engraftment in th e human gut following fecal microbiota transplantation. Cell Host Microbe. 23(2):229–240. 29447696 10.1016/j.chom.2018.01.003 PMC8318347

[15] Walter J , Ley R. 2011. The human gut microbiome: ecology and recent evolutionary changes. Ann Rev Microbiol. 65(1):411–429. 21682646 10.1146/annurev-micro-090110-102830

[16] Huttenhower C et al. 2012. Structure, function and diversity of the healthy human microbiome. Nature. 486(7402):207–214. 22699609 10.1038/nature11234 PMC3564958

[17] Methé BA , et al. 2012. A framework for human microbiome research. Nature. 486(7402):215–221. 22699610 10.1038/nature11209 PMC3377744

[18] Gupta VK , et al. 2020. A predictive index for health status using species-level gut microbiome profiling. Nat Commun. 11:4635. 32934239 10.1038/s41467-020-18476-8 PMC7492273

[19] Costello EK , Stagaman K, Dethlefsen L, Bohannan BJM, Relman DA. 2012. The application of ecological theory toward an understanding of the human microbiome. Science. 336(6086):1255–1262. 22674335 10.1126/science.1224203 PMC4208626

[20] Miller ET , Svanback R, Bohannan BJM. 2018. Microbiomes as metacommunities: understanding host-associated microbes through metacommunity ecology. Trend Ecol Evol. 33(12):926–935. 10.1016/j.tree.2018.09.002 30266244

[21] Dominguez-Bello MG , Godoy-Vitorino F, Knight R, Blaser MJ. 2019. Role of the microbiome in human development. Gut. 68(6):1108–1114. 30670574 10.1136/gutjnl-2018-317503 PMC6580755

[22] Belkaid Y , Hand T. 2014. Role of the microbiota in immunity and inflammation. Cell. 157(1):121–141. 24679531 10.1016/j.cell.2014.03.011 PMC4056765

[23] Costea PI , et al. 2018. Enterotypes in the landscape of gut microbial community composition. Nat Microbiol. 3(1):8–16. 29255284 10.1038/s41564-017-0072-8 PMC5832044

[24] Snijders TAB . 2011. Statistical models for social networks. Ann Rev Sociol. 37(1):131–153.

[25] Vallès-Català T , Peixoto TP, Sales-Pardo M, Guimerà R. 2018. Consistencies and inconsistencies between model selection and link prediction in networks. Phys Rev E. 97:062316. 30011606 10.1103/PhysRevE.97.062316

[26] Guimerà R , Sales-Pardo M. 2009. Missing and spurious interactions and the reconstruction of complex networks. Proc Natl Acad Sci USA. 106(52):22073–22078. 20018705 10.1073/pnas.0908366106 PMC2799723

[27] Peixoto TP . 2019. Bayesian stochastic blockmodeling. In: Doreian P, Batagelj V, Ferligoj A, editors. Advances in network clustering and blockmodeling, New York (NY): Wiley.

[28] Godoy-Lorite A , Guimerà R, Moore C, Sales-Pardo M. 2016. Accurate and scalable social recommendation using mixed-membership stochastic block models. Proc Natl Acad Sci USA. 113:14207–14212. 27911773 10.1073/pnas.1606316113 PMC5167156

[29] Patterson BD , Atmar W. 1986. Nested subsets and the structure of insular mammalian faunas and archipelagos. Biol J Linn Soc. 28(1-2):65–82.

[30] Mariani MS , Ren ZM, Bascompte J, Tessone CJ. 2019. Nestedness in complex networks: observation, emergence, and implications. Phys Rep. 813:1–90.

[31] Poulin R , Valtonen ET. 2001. Nested assemblages resulting from host size variation: the case of endo parasite communities in fish hosts. Int J Parasitol. 31(11):1194–1204. 11513888 10.1016/s0020-7519(01)00262-4

[32] Bascompte J , Jordano P, Melián CJ, Olesen JM. 2003. The nested assembly of plant-animal mutualistic networks. Proc Natl Acad Sci USA. 100(16):9383–9387. 12881488 10.1073/pnas.1633576100 PMC170927

[33] Warburton EM , Van Der Mescht L, Khokhlova IS, Krasnov BR, Vonhof MJ. 2018. Nestedness in assemblages of helminth parasites of bats: a function of geography, environment, or host nestedness?. Parasitol Res. 117:1621–1630. 29594347 10.1007/s00436-018-5844-4

[34] Truong DT , et al. 2015. MetaPhlAn2 for enhanced metagenomic taxonomic profiling. Nat Methods. 12:902–903. 26418763 10.1038/nmeth.3589

[35] Airoldi EM , Blei DM, Fienberg SE, Xing EP. 2008. Mixed membership stochastic blockmodels. J Mach Learn Res. 9(2008):1981–2014. 21701698 PMC3119541

[36] Bastolla U , et al., 2009. The architecture of mutualistic networks minimizes competition and increases biodiversity. Nature. 458(7241):1018–1020. 19396144 10.1038/nature07950

[37] Liu W , et al. 2016. Unique features of ethnic mongolian gut microbiome revealed by metagenomic analysis. Sci Rep. 6:34826. 27708392 10.1038/srep34826 PMC5052615

[38] Qin N , et al., 2014. Alterations of the human gut microbiome in liver cirrhosis. Nature. 513:59. 25079328 10.1038/nature13568

[39] Schirmer M , et al. 2016. Linking the human gut microbiome to inflammatory cytokine production capacity. Cell. 167(4):1125–1136. 27814509 10.1016/j.cell.2016.10.020 PMC5131922

[40] Lloyd-Price J , et al. 2017. Strains, functions and dynamics in the expanded Human Microbiome Project. Nature. 550:61. 28953883 10.1038/nature23889 PMC5831082

[41] Zeevi D , et al. 2015. Personalized nutrition by prediction of glycemic responses. Cell. 163(5):1079–1094. 26590418 10.1016/j.cell.2015.11.001

[42] García-Jiménez B , Wilkinson MD. 2019. Robust and automatic definition of microbiome states. PeerJ. 7:e6657. 30941274 10.7717/peerj.6657 PMC6440462

[43] Arumugam M , et al. 2011. Enterotypes of the human gut microbiome. Nature. 473(7346):174–180. 21508958 10.1038/nature09944 PMC3728647

[44] Knights D , Costello EK, Knight R. 2011. Supervised classification of human microbiota. FEMS Microbiol Rev. 35(2):343–359. 21039646 10.1111/j.1574-6976.2010.00251.x

[45] Holmes I , Harris K, Quince C. 2012. Dirichlet multinomial mixtures: generative models for microbial metagenomics. PLoS ONE. 7(2):e30126. 22319561 10.1371/journal.pone.0030126 PMC3272020

[46] Poux-Médard G , Cobo-Lopez S, Duch J, Guimerà R, Sales-Pardo M. 2021. Complex decision-making strategies in a stock market experiment explained as the combination of few simple strategies. EPJ Data Sci. 10(1):26.

[47] Cook RR , Quinn JF. 1995. The influence of colonization in nested species subsets. Oecologia. 102:413–424. 28306884 10.1007/BF00341353

[48] Perry G , Rodda GH, Fritts TH, Sharp TR. 1998. The lizard fauna of Guam’s fringing islets: island biogeography, phylogenetic history, and conservation implications. Glob Ecol Biogeogr Lett. 8:353–365.

[49] Hastings A , Cai W, Snyder J, D’Souza RM. 2020. Mutualistic networks emerging from adaptive niche-based interactions. Nat Commun. 11:5470. 33122629 10.1038/s41467-020-19154-5 PMC7596068

[50] MacArthur R , Levins R. 1964. Competition, habitat selection, and character displacement in a patchy environment. Proc Natl Acad Sci USA. 51:1207. 14215645 10.1073/pnas.51.6.1207 PMC300237

[51] Dykhuizen D , Davies M. 1980. An experimental model: bacterial specialists and generalists competing in chemostats. Ecology. 61(5):1213–1227.

[52] Grice EA , et al. 2009. Topographical and temporal diversity of the human skin microbiome. Science. 324(5931):1190–1192. 19478181 10.1126/science.1171700 PMC2805064

[53] Bascompte J , Jordano P. 2007. Plant-animal mutualistic networks: the architecture of biodiversity. Ann Rev Ecol Evol Syst. 38:567–593.

[54] Bronstein JL . Mutualism. Oxford: Oxford University Press.

[55] Suweis S , Simini F, Banavar JR, Maritan A. 2013. Emergence of structural and dynamical properties of ecological mutualistic networks. Nature. 500:449–452. 23969462 10.1038/nature12438

[56] Thébault E , Fontaine C. 2010. Stability of ecological communities and the architecture of mutualistic and trophic networks. Science. 329(5993):853–856. 20705861 10.1126/science.1188321

[57] Allesina S , Tang S. 2012. Stability criteria for complex ecosystems. Nature. 438:205–208. 10.1038/nature10832 22343894

[58] Pascual-García A , Bastolla U. 2017. Mutualism supports biodiversity when the direct competition is weak. Nat Commun. 8:14326. 28232740 10.1038/ncomms14326 PMC5512850

[59] Haque SZ , Haque M. 2017. The ecological community of commensal, symbiotic, and pathogenic gastrointestinal microorganisms - an appraisal. Clin Exp Gastroenterol. 10:91–103. 28503071 10.2147/CEG.S126243 PMC5426469

[60] Suweis S , Simini F, Banavar JR, Maritan A. 2008. Grabitske HA, Slavin JL. Low-digestible carbohydrates in practice. J Am Diet Assoc. 108:1677–1681. 18926133 10.1016/j.jada.2008.07.010

[61] Sung J , et al. 2017. Global metabolic interaction network of the human gut microbiota for context-specific community-scale analysis. Nat Commun. 8:15393. 28585563 10.1038/ncomms15393 PMC5467172

[62] Wright DH , Patterson BD, Mikkelson GM, Cutler A, Atmar W. 1997. A comparative analysis of nested subset patterns of species composition. Oecologia. 113:1–20. 28307284 10.1007/s004420050348

[63] Fernández-Juricic E . 2002. Can human disturbance promote nestedness? A case study with breeding birds in urban habitat fragments. Oecologia. 131:269–278. 28547695 10.1007/s00442-002-0883-y

[64] Simberloff DS , Wilson EO. 1969. Experimental zoogeography of islands: the colonization of empty islands. Ecology. 50(2):278–296.

[65] Piechnik DA , Lawler SP, Martinez ND. 2008. Food-web assembly during a classic biogeographic study: species’ “trophic breadth” corresponds to colonization order. Oikos. 117:665–674.

