# Supplementary material for: Stochastic block models reveal a robust nested pattern in healthy human gut microbiomes
Source: PNAS Nexus. 2022 May 23;1(3):pgac055. doi: 10.1093/pnasnexus/pgac055 (PMC9896942; doi:10.1093/pnasnexus/pgac055)
Supplement: pgac055_Supplemental_Files [file pgac055_supplemental_files.zip › PNASNEXUS-PNASNEXUS-2021-00125-s04.pdf]

Supplementary Material: Stochastic block models reveal a robust nested pattern in healthy gut microbiomes

S. Cobo-López[1], V. K. Gupta[2,3], Jaeyun Sung[2,3,4], R. Guimerà[1,5,*], and M. Sales-Pardo[1,*]

[1]*Departament d'Enginyeria Química, Universitat Rovira i Virgili, Tarragona, Catalonia, Spain*

[2]*Microbiome Program, Center for Individualized Medicine, Mayo Clinic, Rochester, MN, USA*

[3]*Division of Surgery Research, Department of Surgery, Mayo Clinic, Rochester, MN, USA*

[4]*Division of Rheumatology, Department of Medicine, Mayo Clinic, Rochester, MN, USA*

[5]*Institució Catalana de Recerca i Estudis Avançats, Barcelona, Catalonia, Spain*

[*] *To whom correspondence should be addressed: roger.guimera@urv.cat, marta.sales@urv.cat*

I. SUPPLEMENTARY TABLES

Table S1 – file: Supplementary_Table_1.xlsx

For the microbe species in the datasets we analyze we provide the classification for different taxonomic ranks.

We use the following key for datasets:

- S-8 – Liu *et al.*
- V-10 – Qin *et al*
- V-22 – Schirmer *et al.*
- V-23_24 – Huttenhower *et al.*/Lloyd-Price *et al.*
- V-25 – Zeevi *et al.*

Table S2 – file: Supplementary_Table_2.xlsx

For each species we analyze we provide:

- S.N. (Species Number - internal id)
- Species Name (MetaPhlAn)
- Species Name (on NCBI)
- rank in ordered matrices for each dataset (same key as in Supplementary Table 1)
- Mean (mean rank) across datasets
- Normalized Mean (rank) across datasets
- SEM (standard deviation of mean rank)
- Normalized SEM (standard deviation of the normalized mean rank)
- Median Genome Size
- Minimum Genome Size
- Maximum Genome Size

- SD_genome size (standard deviation of genome size)
- Median Protein #
- Minimum Protein #
- Maximum Protein #
- SD_Protein # (standard deviation of the number of proteins)

Table S3 – file: Supplementary_Table_3.xlsx

For each microbe species in the datasets we analyze we provide:

- Organism/Name
- Size (Mb)
- Proteins
- BioSample Accession
- Assembly Accession
- Strain
- Species Name

II. METRICS FOR PREDICTION POWER ASSESSMENT

a. Accuracy is the fraction of correctly predicted data points (true positives and true negatives) out of the total (see Fig. S1). It is a particularly useful metric if the number of positives is comparable to the number of negatives. However, that is not often the case, and thus accuracy can be a misleading metric. For instance, in very sparse datasets (OTU matrices with a large proportion of entries equal to 0), even the simplest baseline could have very high accuracy rates. To prevent this, we use the recall and precision.

b. Recall is the fraction of true positives out of the total existing ones in the test dataset. However, the recall on its own is not completely informative; we could come up with a method that simply overclassifies data points as positives. Therefore, the recall would be very high, but many predictions would be wrong due to a biased tendency to missclassify negatives (0) as positives (1).

c. Precision is the fraction of true positives out of all predicted positives.

These three metrics are complementary and provide an overall picture of the performance of a method in a prediction task. Note that we show the aggregated accuracy, precision and recall. That is, the predictions for the whole OTU matrix.

Also note that because recall and precision can only be measured for binary outcomes, we reduce the problem to predicting whether a microbial species is present or not in a host. To that end, we merge the low (II) and high (III) abundances into a single 'non-negligible' category. For the SBM approach all the results we show correspond to $K = 10$, $L = 20$.

III. RELATIONSHIP BETWEEN PREDICTABILITY AND ENTROPY IN HOST-SPECIFIC CROSS-VALIDATION EXPERIMENTS

As we have already mentioned, our prediction experiments can help us address questions of clinical relevance such as whether there are hosts or microbes whose relative abundances are easier to predict. Easy to predict hosts/microbes are those that follow an identifiable pattern, whereas those hosts/microbes hard to predict fall out of the norm.

First of all, we observe that while the majority of both hosts and microbes are highly predictive, they do display a large variability, with some hosts and microbes being very unpredictable (Supplementary Figs. 2-6).

To investigate if there is a relationship between the predictability and the model parameters,

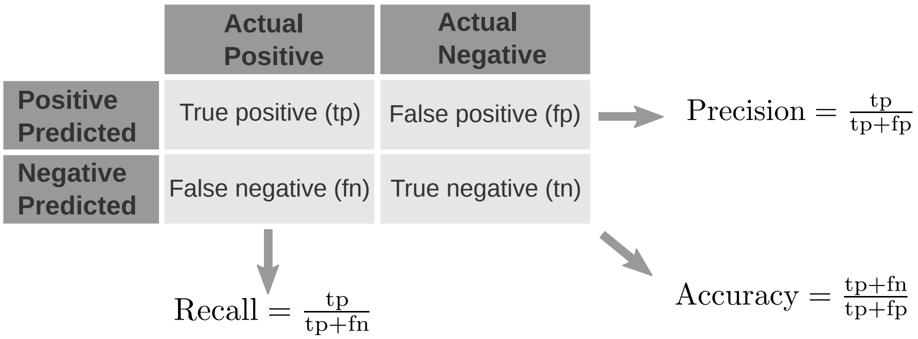

FIG. S1. **Contingency matrix of a classification method** Elements in the diagonal are correctly predicted data points (true positives and true negatives). Off-diagonal elements represent missclassified data points either as false positives or false negatives. The precision measures the ratio of true positives out of all predicted positives, recall measures the fraction of true positives out of the total positives, and accuracy measures the ratio of correctly predicted observations.

we look at how group membership vectors ($\boldsymbol{\theta}$ and $\boldsymbol{\eta}$) are distributed across groups. We measure the distribution of group memberships of each host h and microbe m using the Shannon entropy H_h and H_m of their corresponding membership vector θ_p and η_m, respectively (see next section). Low values of Shannon entropy correspond to membership vectors being concentrated in one or two groups, whereas high values indicate that membership is distributed across many groups.

We find that for hosts and microbes membership vectors are typically distributed among a few groups. We also find that for both hosts and microbes predictability is negatively correlated with Shannon entropy in all datasets (see Figs. S1-5). The statistical significance of the correlation varies, but overall it is consistent across datasets even though it is weaker for the smaller datasets (W. Liu *et al.* and N. Qin *et al.*).

Importantly, individual predictability is typically high even when memberships are distributed across several groups. Therefore having memberships distributed across different groups is a better model for observed microbe relative abundances in hosts than assuming that hosts belong to a single group.

SHANNON ENTROPY

We use the Shannon entropy to measure the extent to which the membership of a microbe or host is mixed across different groups. For each microbe or host, we take its membership vector θ_h,

η_m and compute its Shannon entropy as follows:

$$H_h = -\sum_{i}^{K} \theta_{hi} \log_K(\theta_{hi}) \qquad H_m = -\sum_{j}^{L} \eta_{hj} \log_L(\eta_{hj}), \tag{1}$$

where K and L correspond to the number of groups of hosts and microbes (10 and 20 respectively). The numerical values of the Shannon entropy range from 0 to $1.H = 1$ represents a situation in which a microbe or host belongs to all groups with identical weights, i.e.:

$$\theta_h = [1/K, \ldots, 1/K] \qquad \eta_m = [1/L, \ldots, 1/L], \tag{2}$$

while $H = 0$ means that the host or microbe has its membership concentrated in a single group:

$$\theta_h = [1, 0, \ldots, 0] \qquad \eta_m = [1, 0, \ldots, 0]. \tag{3}$$

In the context of information theory, these extreme situations correspond to the minimum and maximum information scenarios, respectively.

SUPPLEMENTARY FIGURES

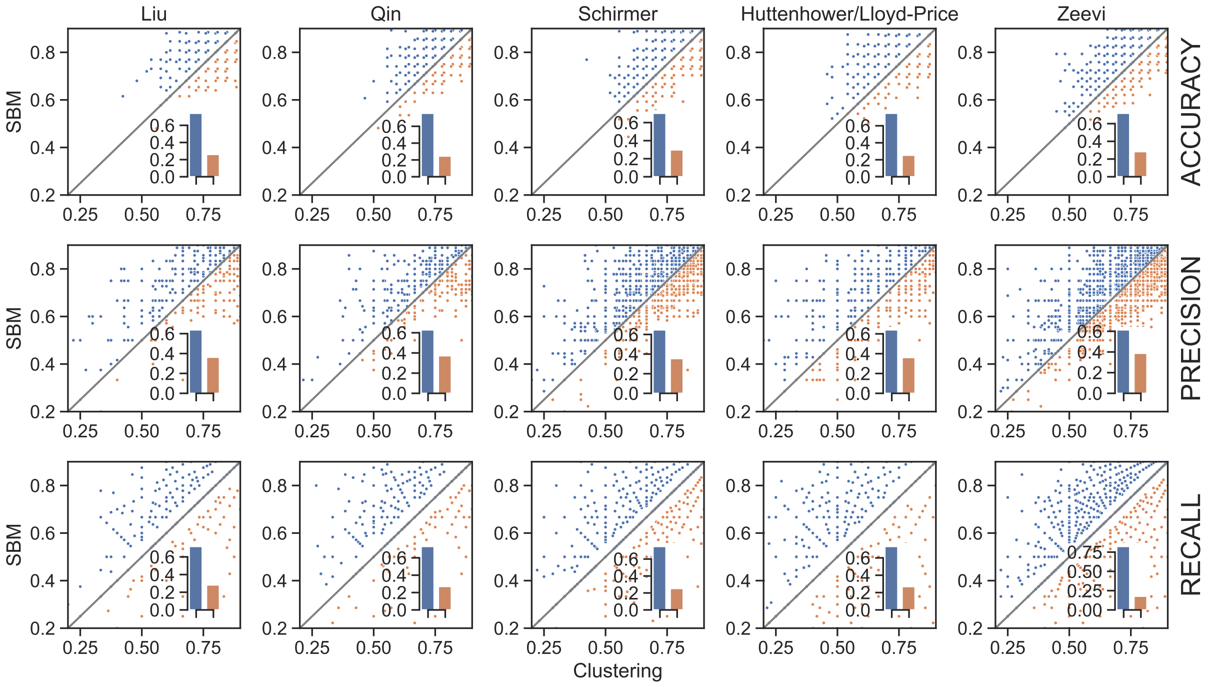

FIG. S2. Comparisons between the predictive performances of the stochastic block model (SBM) and of the agglomerative clustering approach we consider. We consider three performance metrics (overall accuracy [top row], precision [middle row], and recall [bottom row]) to report how well the held-out microbial relative abundance categories in individual hosts can be predicted. Each scatter-plot corresponds to a dataset (plots in the same column correspond to the same dataset) and to a specific performance metric. Each point in a scatter-plot represents a host and a fold. The diagonal line (in grey) indicates equal performance of both approaches. Blue points correspond to hosts for which the SBM outperforms the clustering approach, whereas orange points correspond to hosts for which the clustering approach outperforms the SBM approach. Because points in the scatter plot overlap, it is difficult to estimate visually how often the SBM outperforms the clustering approach. To accommodate for this, the inset in each figure shows the fraction of the study's tested hosts whose abundances are better predicted by the SBM (blue) and clustering (orange) approaches.

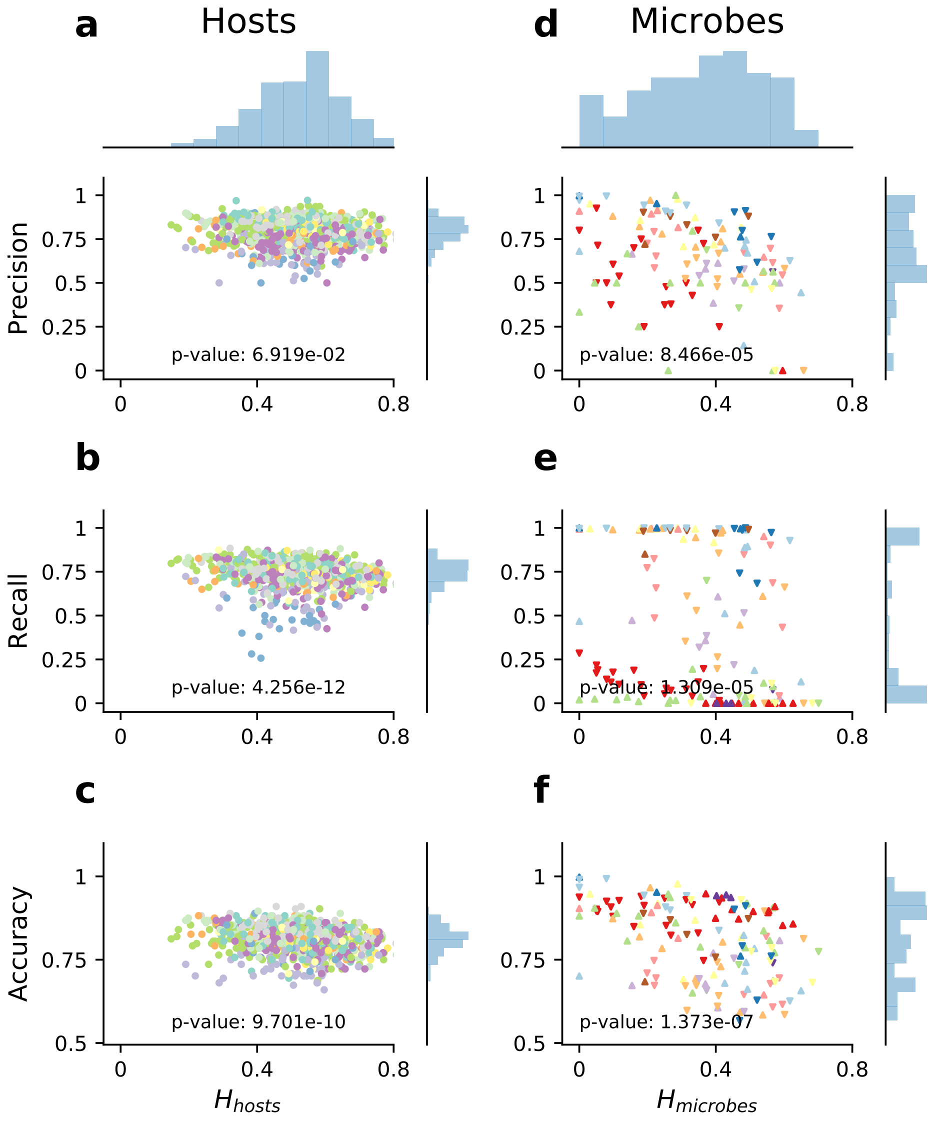

FIG. S3. **Individual predictability for Zeeve *et al.*** We show the predictive performance of the SBM on individual hosts and microbes vs the Shannon Entropy of their membership vectors. The predictive performance is measured using the precision **[a,d]**, recall **[b,e]**, and accuracy **[c,f]**. In the plots, each colored square/triangle represents an individual host/microbe, while color and shape (if applies) correspond to its top membership, that is the group for which the membership weight of a host is the largest. We also show the distributions of Shannon Entropies and corresponding predictive metrics. In general, we observe a statistically significant negative correlation between Shannon Entropies and predictive metrics.

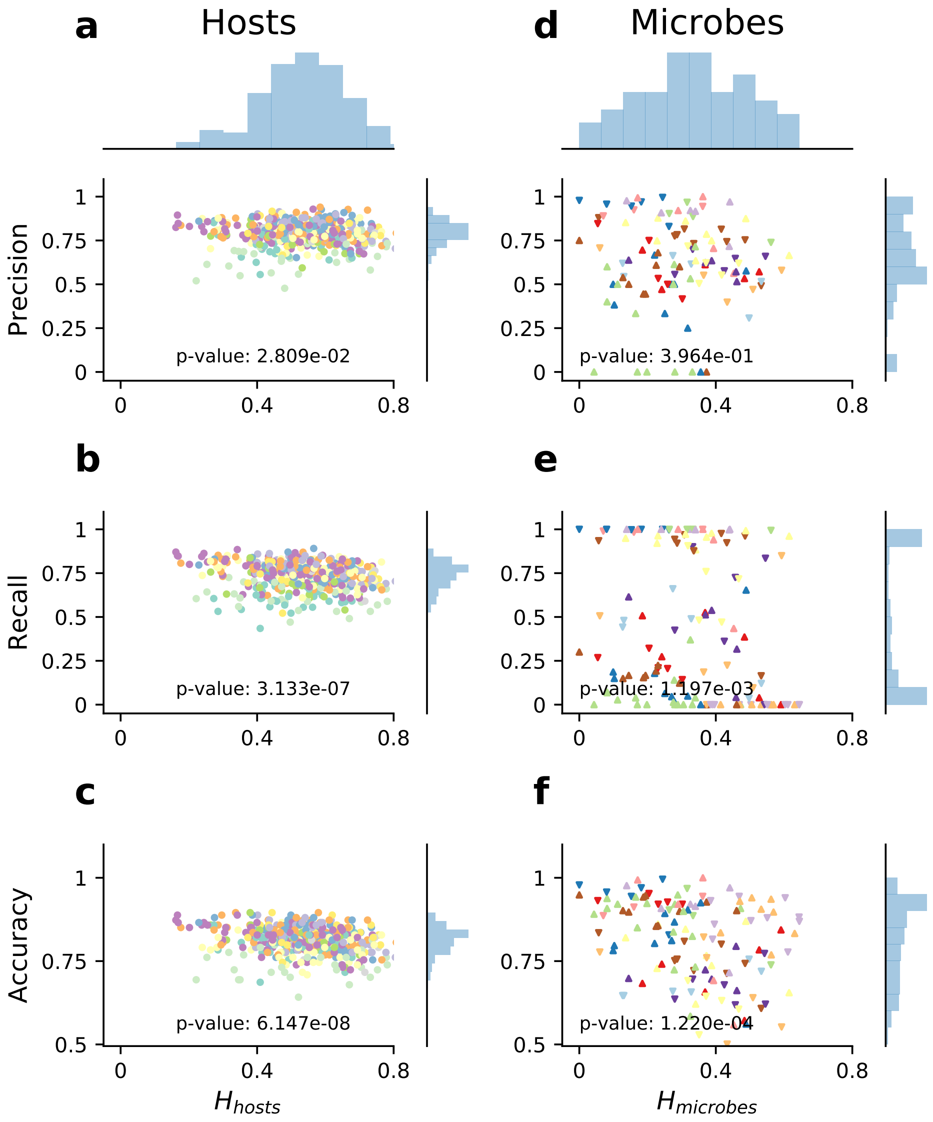

FIG. S4. **Individual predictability for the dataset of Schirmer *et al.*** We show the predictive performance of the SBM on individual hosts and microbes vs the Shannon Entropy of their membership vectors. The predictive performance is measured using the precision **[a,d]**, recall **[b,e]**, and accuracy **[c,f]**. In the plots, each colored square/triangle represents an individual host/microbe, while color and shape (if applies) correspond to its top membership, that is the group for which the membership weight of a host is the largest. We also show the distributions of Shannon Entropies and corresponding predictive metrics. In general, we observe a statistically significant negative correlation between Shannon Entropies and predictive metrics.

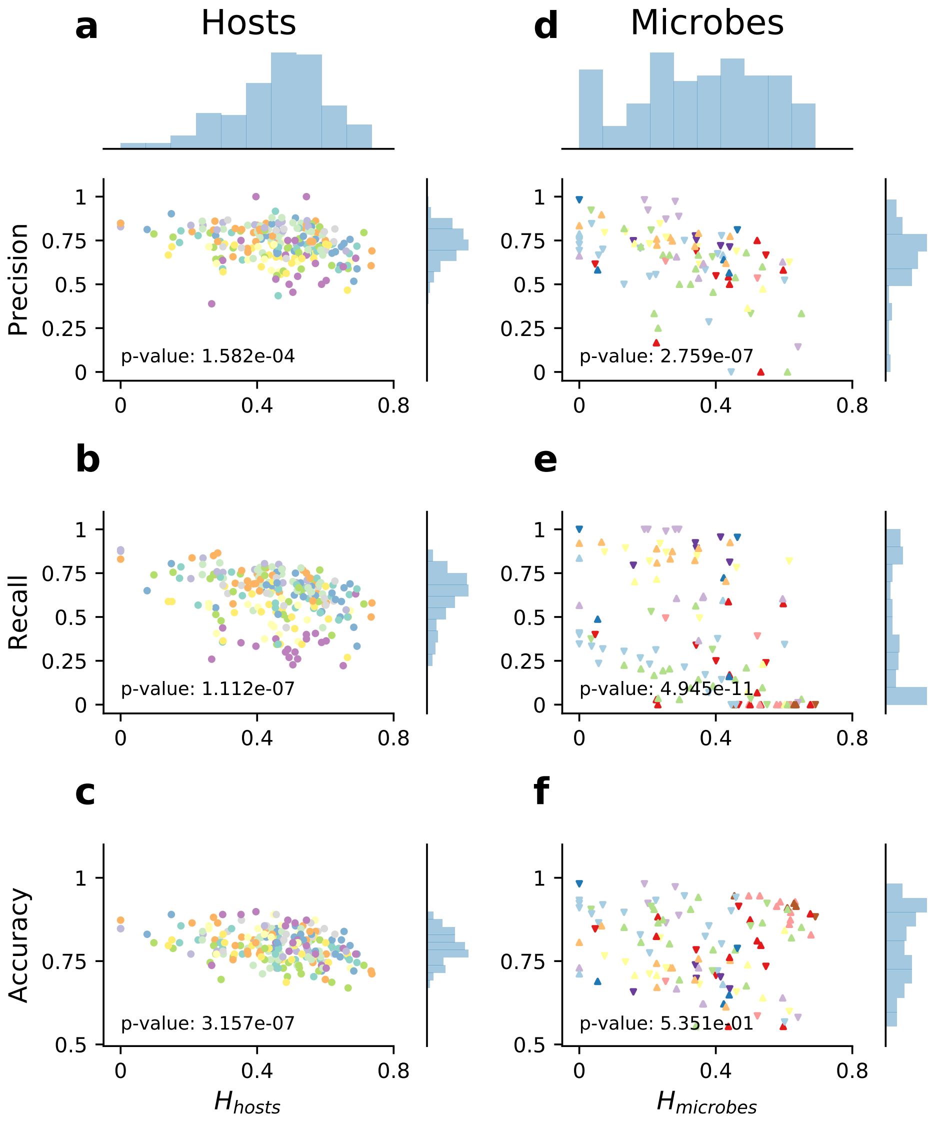

FIG. S5. **Individual predictability for the dataset of C.Huttenhower *et al.* and J Lloyd-Price *et al.*** We show the predictive performance of the SBM on individual hosts and microbes vs the Shannon Entropy of their membership vectors. The predictive performance is measured using the precision **[a,d]**, recall **[b,e]**, and accuracy **[c,f]**. In the plots, each colored square/triangle represents an individual host/microbe, while color and shape (if applies) correspond to its top membership, that is the group for which the membership weight of a host is the largest. We also show the distributions of Shannon Entropies and corresponding predictive metrics. In general, we observe a statistically significant negative correlation between Shannon Entropies and predictive metrics.

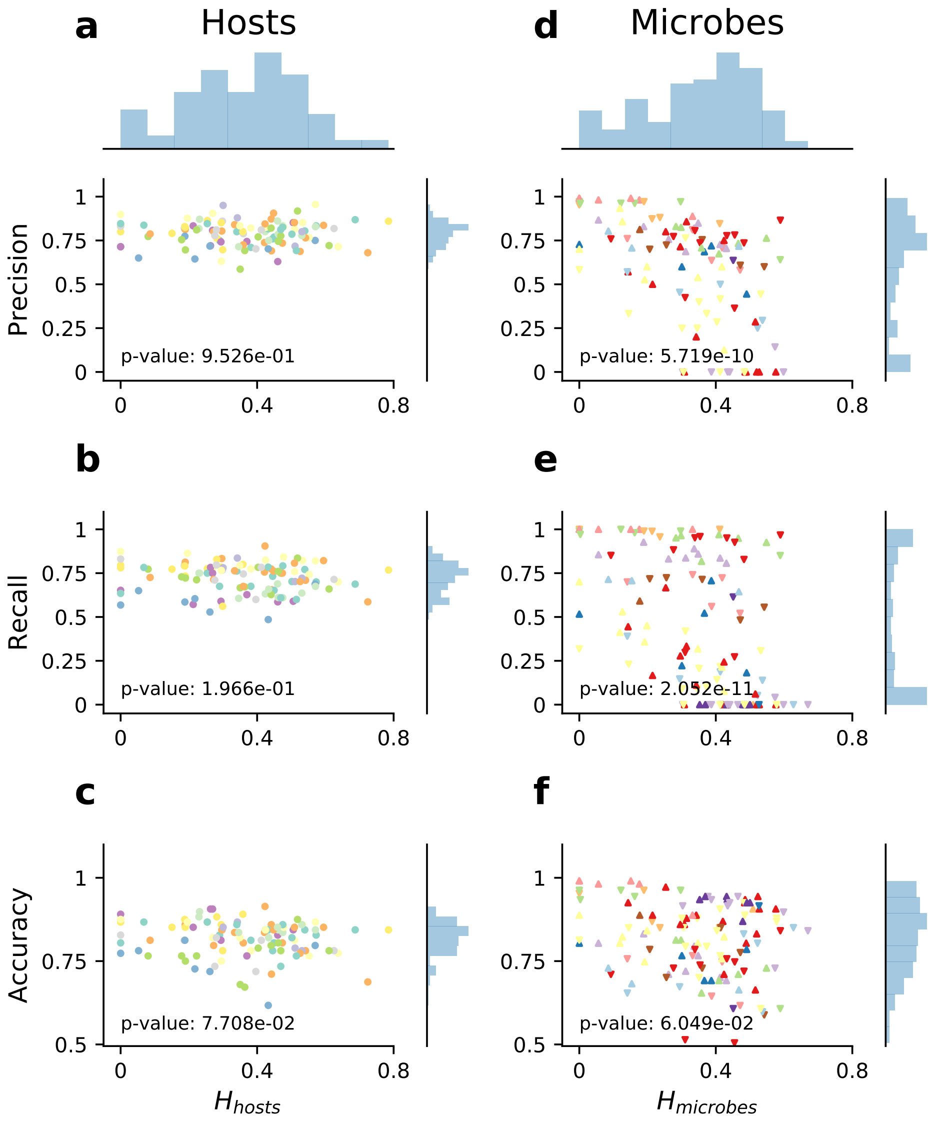

FIG. S6. **Individual predictability for the dataset of W. Liu *et al.*** We show the predictive performance of the SBM on individual hosts and microbes vs the Shannon Entropy of their membership vectors. The predictive performance is measured using the precision **[a,d]**, recall **[b,e]**, and accuracy **[c,f]**. In the plots, each colored square/triangle represents an individual host/microbe, while color and shape (if applies) correspond to its top membership, that is the group for which the membership weight of a host is the largest. We also show the distributions of Shannon Entropies and corresponding predictive metrics. In general, we observe a statistically significant negative correlation between Shannon Entropies and predictive metrics.

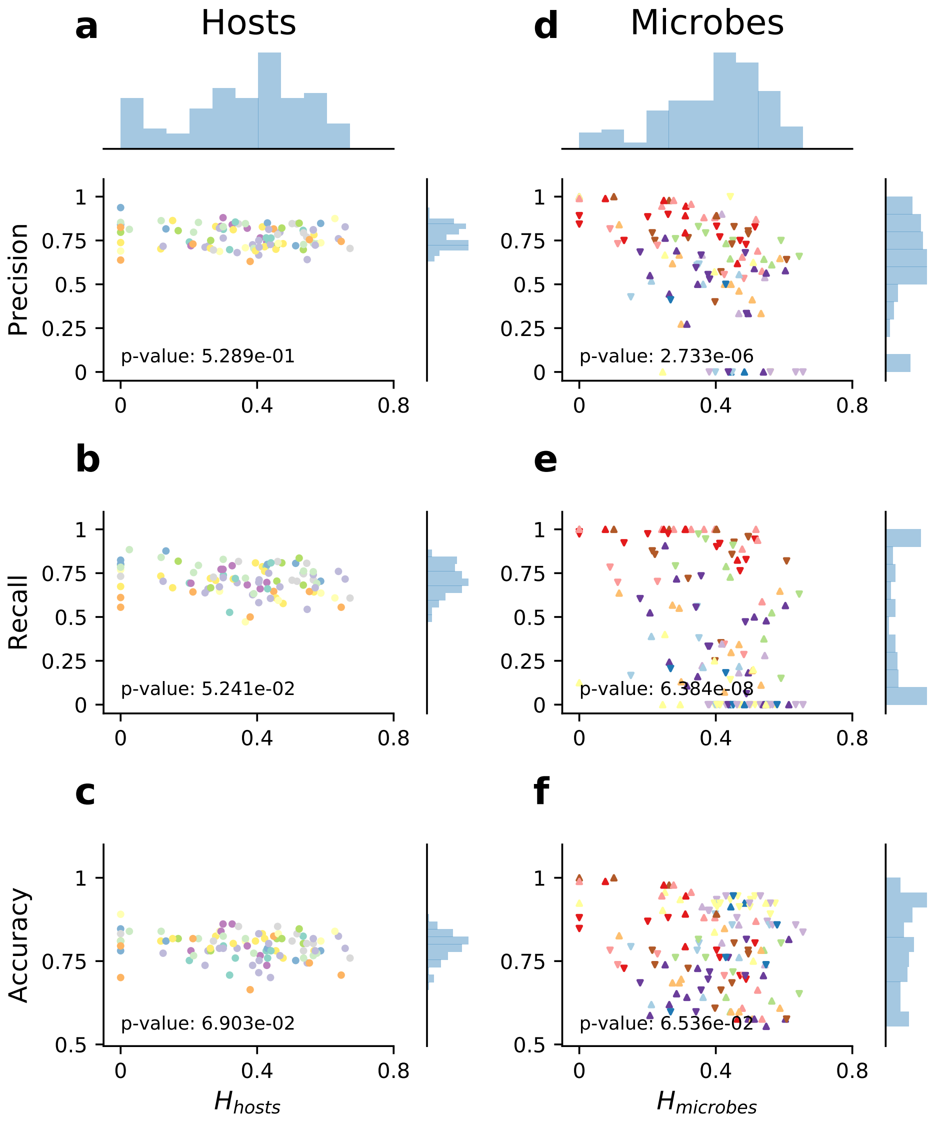

FIG. S7. **Individual predictability for dataset of N. Qin *et al.*** We show the predictive performance of the SBM on individual hosts and microbes vs the Shannon Entropy of their membership vectors. The predictive performance is measured using the precision **[a,d]**, recall **[b,e]**, and accuracy **[c,f]**. In the plots, each colored square/triangle represents an individual host/microbe, while color and shape (if applies) correspond to its top membership, that is the group for which the membership weight of a host is the largest. We also show the distributions of Shannon Entropies and corresponding predictive metrics. In general, we observe a statistically significant negative correlation between Shannon Entropies and predictive metrics.

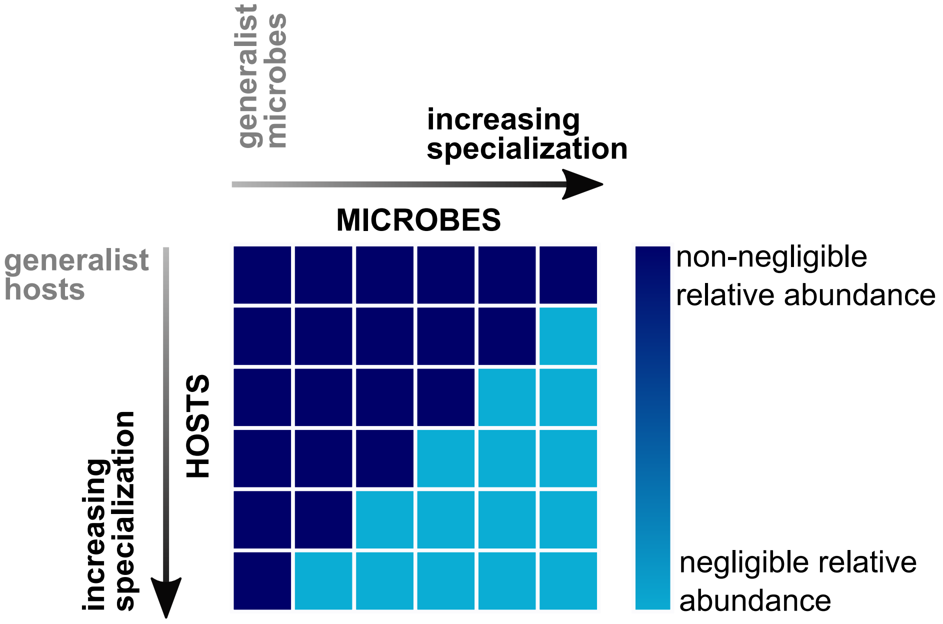

FIG. S8. **Perfectly nested relative abundance matrix.** Specialist microbes are present in the microbiome of generalist hosts, which are a subset of the hosts in which generalist microbes are present. Specialist host only have generalist microbes in their microbiomes.

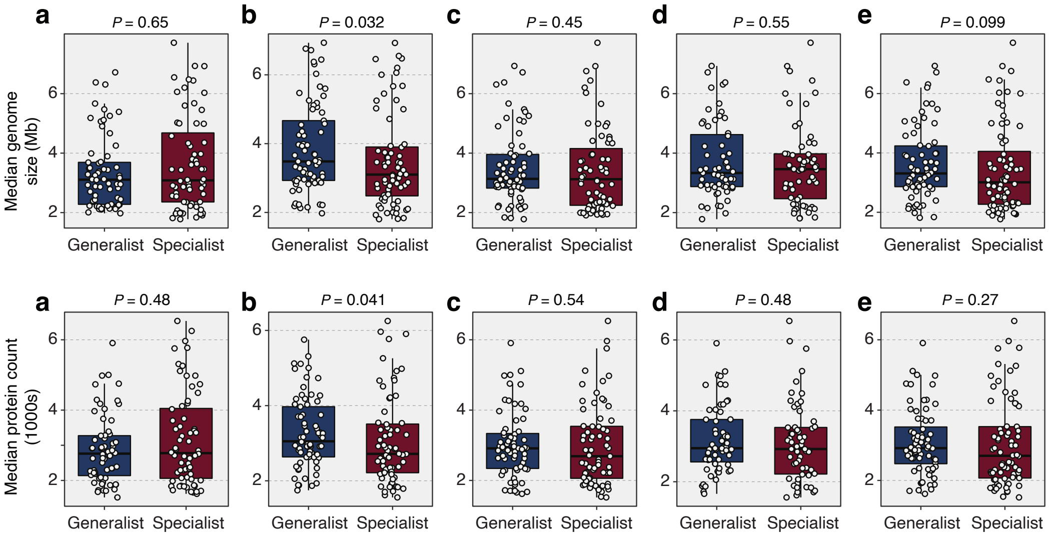

FIG. S9. **Relationship between ecological microbial roles and genome size** We show the differences in genome sizes (top panel) and protein coding gene counts (bottom panel) between microbial species of generalist microbes (top 50% ranks) and specialist microbes (bottom 50% ranks). In four of the study datasets (**a, c-e**), we do not observe any significant difference (Two-sided Mann–Whitney U test, $P > 0.05$) in median genome sizes (upper panel) and count of protein coding genes (lower panel) between generalist (top 50% ranks) and specialist (bottom 50% ranks) microbial species. Only in Qin et al. 2014 study dataset (**b**) we observe a significant difference in median genome sizes and in count of protein coding genes between generalist and specialist microbial species (Two-sided Mann–Whitney U test, $P < 0.05$). We use standard box-and-whisker plots (e.g., center line, median; box limits, upper and lower quartiles; whiskers, $1.5 \times$ interquartile range; circles, outliers) to depict groups of numerical data. Study datasets: **a)** Liu et al. 2016; **b)** Qin et al. 2014; **c)** Schirmer et al. 2016; **d)** Huttenhower et al., 2012 and J. Lloyd-Price et al. 2017; **e)** Zeevi et al. 2015.

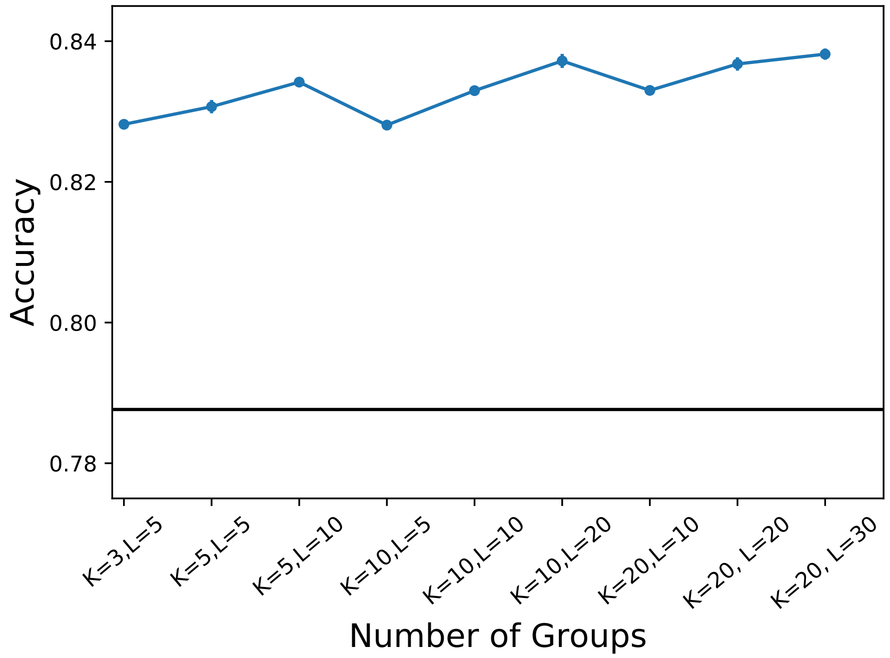

FIG. S10. **Selection of K and L** We compute the average predictive accuracy for different values of K and L. We pool all five datasets together and do a 5-fold cross validation for testing the model performance. In the plot, each dot corresponds to the mean over all five splits and error bars correspond to the standard error of the mean. In some cases, error bars are smaller than symbol size. The black solid line shows the performance of a baseline that always predicts a zero abundance. Note that for $K > 10$ and $L > 20$ there is no improvement in accuracy with respect to $K = 10$, $L = 20$.

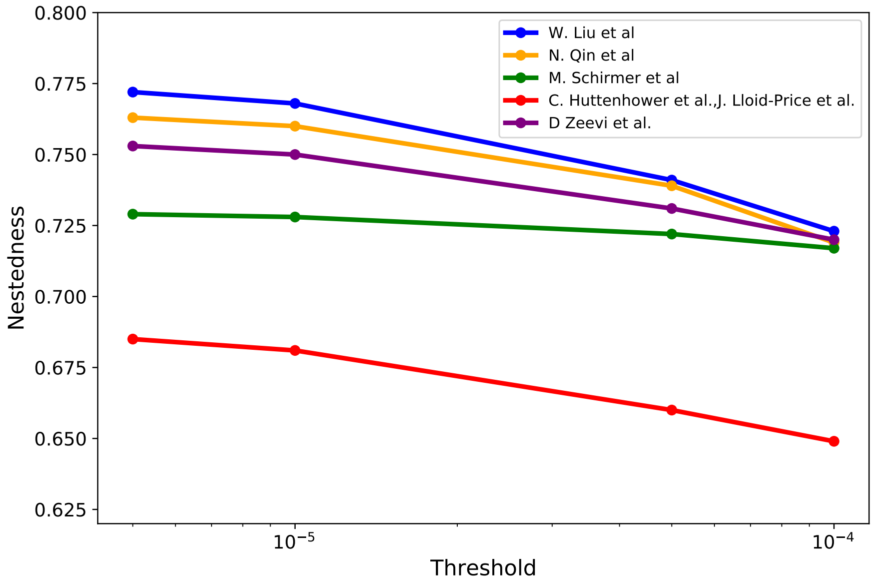

FIG. S11. **Effect of threshold on nestedness** For the different gut microbiome datasets analyzed in our study, we categorized the relative abundances into two discrete classes: 'non-negligible' for microbes of relative abundance greater or equal to a given threshold; and 'negligible' for microbes of relative abundance smaller than the threshold. We then computed the nestedness of each dataset's relative abundance matrix for different threshold values. Each point corresponds to a nestedness value, and the connecting lines are there to guide the eye. Different colors correspond to different datasets. Of note, as we lower the threshold starting from the one used in our analysis (1×10^{-4}), the nestedness gradually increases but does not fluctuate in any unpredictable manner. Hence, the nestedness is robust to changes in the binary discretization threshold.
